# The dynamics of intracellular water constrains glycolytic oscillations in *Saccharomyces cerevisiae*

**DOI:** 10.1038/s41598-017-16442-x

**Published:** 2017-11-24

**Authors:** Henrik S. Thoke, Sigmundur Thorsteinsson, Roberto P. Stock, Luis A. Bagatolli, Lars F. Olsen

**Affiliations:** 1 Center for Biomembrane Physics (MEMPHYS), Odense M, Denmark; 2 Institute for Biochemistry and Molecular Biology, University of Southern Denmark, Campusvej 55, DK5230 Odense M, Denmark; 3 Yachay EP and Yachay Tech, Yachay City of Knowledge, 100650 Urcuquí-Imbabura, Ecuador

## Abstract

We explored the dynamic coupling of intracellular water with metabolism in yeast cells. Using the polarity-sensitive probe 6-acetyl-2-dimethylaminonaphthalene (ACDAN), we show that glycolytic oscillations in the yeast *S. cerevisiae* BY4743 wild-type strain are coupled to the generalized polarization (GP) function of ACDAN, which measures the physical state of intracellular water. We analysed the oscillatory dynamics in wild type and 24 mutant strains with mutations in many different enzymes and proteins. Using fluorescence spectroscopy, we measured the amplitude and frequency of the metabolic oscillations and ACDAN GP in the resting state of all 25 strains. The results showed that there is a lower and an upper threshold of ACDAN GP, beyond which oscillations do not occur. This critical GP range is also phenomenologically linked to the occurrence of oscillations when cells are grown at different temperatures. Furthermore, the link between glycolytic oscillations and the ACDAN GP value also holds when ATP synthesis or the integrity of the cell cytoskeleton is perturbed. Our results represent the first demonstration that the dynamic behaviour of a metabolic process can be regulated by a cell-wide physical property: the dynamic state of intracellular water, which represents an emergent property.

## Introduction

It is well known that many cellular metabolic and signalling processes exhibit temporal oscillations. Examples of such oscillations are circadian rhythms^1^, the cell cycle^2^, oscillations in cell signalling^3^, intracellular calcium and metabolism in pancreatic *β*-cells^4^ and oscillations in yeast and plant metabolism^5^. Many efforts have been made to understand the generative principles underlying oscillatory cellular activities, and most of the proposals have been grounded in a general framework based on mass action kinetics and dilute solution theory. However, a few studies devoted to measurements of properties such as heat flux^6^, electrical conductivity^7^ and intracellular pH^8^ indicate that factors other than simple diffusion and enzyme kinetics may contribute to oscillations mechanisms. In this study, we present experiments that suggest another controlling factor, specifically the dynamic state of intracellular water. To address the role of water in the dynamics of metabolic oscillations, we made use of oscillating glycolysis in *S. cerevisiae*, a well-established and characterized model system.

The amplitude and frequency of oscillations in glycolysis (as measured by NADH production) in yeast and in other organisms have been studied now for six decades^9,10^. Nevertheless, despite many efforts to create realistic models based on what is known about the enzymes that catalyse the individual reactions in this pathway, the precise mechanisms underlying these oscillations remain unclear. Most of these models build on the regulatory properties of glycolytic enzymes^11,12^, and a few also take into account the fact that glycolysis is, by its very nature, an autocatalytic process with respect to ATP^13–15^. In a previous study^16^ we presented evidence of tight coupling between such metabolic oscillations of glycolysis and intracellular water dynamics. We established that water dipolar relaxation - a measure of the freedom of water dipoles to reorient in response to changes in dipole moments - oscillates with glycolysis and is in phase with intracellular ATP levels. Furthermore, this phenomenon was shown to be scale-invariant from the subcellular level to ensembles of synchronized cells. So far, none of the existing models of glycolytic oscillations can account for this coupling between the physical state of water and a metabolic process.

Comprising 70% of the weight of the cell, water constitutes the most abundant cellular component. Its unusual properties as a polar solvent are known to influence essentially all biological processes. Although the state of intracellular water is traditionally considered to be comparable to the liquid state, this may not be a realistic scenario when molecular crowding of the cytoplasm is taken into account^17–21^. In fact, the state and dynamics of intracellular water - and its impact on cell physiology - is still very much under debate^21,22^. Recently, it has become evident that there is a gap between the current view of metabolism and its regulation (as gleaned from studies of enzymes in dilute solutions) and the properties of these same enzymes in the crowded environment of a cell^23,24^. Crowding is understood as the situation in which solutes, by their nature and concentration, alter the intensive (or bulk) properties of the aqueous environment, i.e., viscosity, density and permittivity. In some cases, molecular crowding is conceptualized as an “excluded volume effect”^24^. This view, however, entails that crowding has no significant effect on cell-wide physicochemical properties of water. Whereas in most studies of macromolecular crowding and confinement in cells, water is treated as a dielectric continuum^25^, recent studies using molecular dynamics simulations have shown that the mobility of individual water molecules is very heterogeneous, and a large portion of intracellular water molecules are essentially immobilized in long-lived water bridges between proteins^21,26,27^. Conflicting views exist regarding the state of intracellular (cytoplasmic) water: some reports^20,28–30^ suggest that most intracellular water is in a gel-like or polarized state, while other reports indicate that intracellular water is essentially like bulk water^31^.

Although the idea has not yet been thoroughly explored in biological contexts, it is not unusual for non-biological oscillating chemical reactions to respond to macromolecular crowding. For example, it is well known that the Belousov-Zhabotinskii (BZ) reaction, when carried out in viscous aqueous solutions containing various polymers, may show oscillations in solution viscosity and volume that are synchronized with oscillations in redox potential^32–35^. Furthermore, it has been demonstrated that synchronization involves reversible aggregations of polymers that themselves depend on the redox state of the metal complex catalysing the BZ reaction^35^ and that the oscillatory behaviour depends on the viscosity of the solution. Analogously, it could be hypothesized that the oscillations in dynamics of water, as observed by oscillations in the fluorescence of the DAN probes^16^, are modulated by reversible changes of macromolecules (e.g., cytoskeleton proteins) in the intracellular space of the yeast cell. Furthermore, these oscillations are in turn synchronized with metabolite oscillations (e.g., ATP) during oscillatory glycolysis. This hypothesis is investigated here.

## Results

### ACDAN GP measures the physical state of water

Adding glucose and potassium cyanide (KCN) to a dense suspension of the yeast *S. cerevisiae* results in glycolytic oscillations on a time scale of 40 s (see Figs S1–S3). These oscillations can be monitored directly as oscillations in NADH fluorescence^9,10^, but other variables, such as various glycolytic intermediates (including ATP)^36,37^, carbon dioxide^38^, intracellular pH and membrane potentials of organelles (mitochondria, vacuoles)^8^, have been shown to oscillate synchronously with NADH. In a recent paper, we showed that the fluorescence of ACDAN and two other dimethylamino naphthalene (DAN) probes, PRODAN and LAURDAN, which are sensitive to the dipolar relaxation of water in their vicinity, also show oscillations^16^. These probes were synthesized by Gregorio Weber in 1979^39^ and were designed to be used as relaxation probes for various biological environments^39,40^.

As mentioned in the Introduction, the main conclusion of our previous study^16^ was that the dynamics of intracellular water are coupled to oscillating glycolysis. However, it was not clear if the oscillations in, for example, ATP were driving the oscillations of water dynamics or if the latter had a direct regulating effect on the occurrence of the oscillations. Therefore, here, we conducted a series of experiments to explore whether a direct correlation between glycolytic oscillations (as measured by NADH fluorescence) and water dynamics, as measured by the ACDAN fluorescence response, could be established. For the latter, we measured ACDAN fluorescence intensity and the generalized polarization (GP) function, defined by equation (S1) in the Supplementary Information. First, we established that ACDAN GP is an appropriate measure of crowding by comparing GP values with other measures of crowding in energy-depleted yeast cells. Previously, Joyner *et al*.^30^ and Munder *et al*.^29^ measured the diffusion constants of various proteins and mRNPs (messenger ribonucleoproteins) in energy-depleted yeast cells. Using different procedures for energy-depletion, both groups observed that diffusion constants were greatly reduced in these cells. In addition, the cells became stiffer. The reduction in diffusion constants was accompanied by a reduction in intracellular pH. Here, we found that the intracellular pH dropped from pH 7.2 to 6.3 after a period of 30 min, and at the same time, the ACDAN GP increased by approximately 0.01 when we employed the glucose-depletion procedure of Joyner *et al*.^30^. It is important to note that the DAN probes are not sensitive to pH^16^, suggesting the influence of other environmental factors. Thus, the ACDAN GP value shows the same trend as the diffusion constants of macromolecules^29,30^, supporting the idea that ACDAN GP responds to the same changes in intracellular environment observed by others^29,30^.

### ACDAN oscillates throughout the cytoplasm

**Figure 1:**
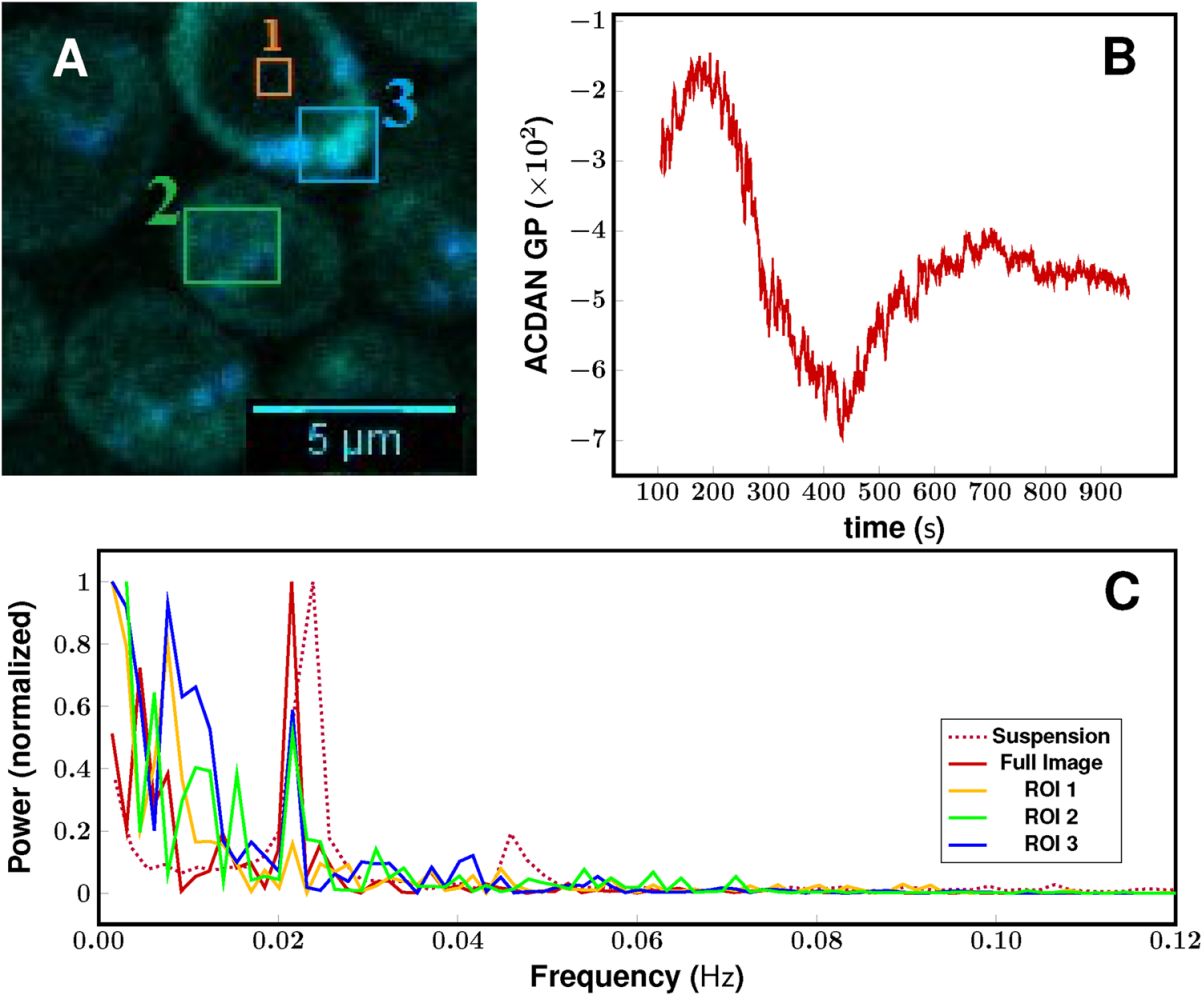
Oscillations in ACDAN GP in resting BY4743 yeast cells. (**A**) Fluorescence images of ACDAN were obtained on an inverted multiphoton excitation fluorescence microscope, with an excitation wavelength for ACDAN at 810 nm. The fluorescent signal was collected through a 63X oil objective, NA 1.4, and split to two detectors filtered by a 520 ± 17.5 nm bandpass filter and a 438 ± 12 nm bandpass filter, respectively. The sampling period was 1200 and the sampling frequency was one image per 0.75 s. (**A**) ROIs were selected such that they corresponded to (1) the central vacuole, (2) an area of high relaxation and (3) an area of low relaxation. (**B**) Time profile of relative ACDAN GP were calculated via Eq. S1. using the mean intensities from the image sequence of the blue and green channels, following addition at time *t* = 0 s of 30 mM glucose and 60 s later 5 mM KCN. The presented data in (**B**) is from the entire field of view (full image). This is conceptually similar to Fig. S1C, albeit the lack of continous stirring tends to shorten the total oscillation length, and cause a slight insignificant decrease in the frequency. (**C**) Power spectra of the intensity profiles at time 200 s–460 s from the full image and selected ROIs in (**A**). ACDAN GP oscillations are seen in every part of the cell, except for in the central vacuole (ROI 1). The dotted line represents the power spectrum of the ACDAN GP measured in a cell suspension.

Next, we measured oscillations in the fluorescence intensity of NADH (Fig. S1A) and ACDAN (Fig. S1B), as well as in the ACDAN GP function (Fig. S1C) in suspensions of the yeast strain BY4743, which is a wild type strain from the Euroscarf systematic deletion project^41^. All three time series showed the same main oscillatory frequency corresponding to an oscillation period of ~40 s as indicated by fast Fourier transform (FTT) analysis (Fig. S1D), confirming our previous results^16^. The oscillations in NADH and ACDAN fluorescence were accompanied by oscillations of many glycolytic intermediates, two of which are shown in Figs S2 and S3. The oscillations in ATP and ACDAN GP shown in Fig. S3 reveal that these two variables oscillate in phase. Furthermore, the phase plot of ACDAN fluorescence versus NADH fluorescence (Fig. S3D) reveals that ACDAN and NADH oscillate 180° out of phase. Fluorescence microscopy of cells labelled with ACDAN (see Fig. 1) demonstrated that the probe is widely distributed throughout the cytoplasm, excluding the central vacuole. Fluorescence emissions in the green and blue regions of the ACDAN spectrum were obtained by splitting the emission into two channels at 438 ± 12 nm and 520 ± 17.5 nm and reveal areas of high and low relaxation within each cell. ACDAN displays very heterogeneous relaxation, as can also be seen in the spectral image (Fig. S4), where different colours correspond to a 10 nm emission interval, similar to that obtained in our prior work^16^. Analysis of the intensity profiles from the two channels in Fig. 1(A) following addition of 30 mM glucose and 5 mM KCN revealed oscillations of ACDAN GP, as shown in Fig. 1(B). Fourier analysis of the time profiles in the different regions of interest, as shown in Fig. 1 (C), indicated that ACDAN GP oscillated with the same period in all regions of interest (ROIs), except for the central vacuole, where oscillations were absent. Figure 1(C) reveals that there is no significant difference in the oscillations frequency between the different regions in a single cell, and billions of cells in a suspension (Fig. 1(C) dotted line). To ensure that the ACDAN signal originated from the cytoplasm, we labelled the cells with both ACDAN and carboxyfluorescein diacetate succinimidyl ester (CFDA-SE), which has been used to measure pH in the cytoplasm^8^ Fig. S5 shows that the two dyes co-localize, demonstrating that ACDAN is present in the cytoplasm. The CFDA fluorescence, calculated as in^8^, revealed an intracellular pH of 7.2 before the addition of glucose and KCN. It has previously been suggested that there is a coupling between oscillations in mitochondrial membrane potential and oscillations in NADH mediated by the mitochondiral adenine nucleotide translocater and ATP syntase^8,42^. To investigate how much of the ACDAN signal originates from the mitochondria, we double-stained cells with ACDAN and MitoTracker Red as shown in Fig. S6. The top row in Fig. S6 suggests some co-localization of the two probes for oscillating yeast cells grown on glucose, as used in the other experiments, and hence that some of the ACDAN signal originates from the mitochondria. However, the number of mitochondria in these cells seems to be small and hence it is only a minor fraction of the total ACDAN signal that stems from the mitochondria. To test if MitoTracker Red really stains mitochondria we grew the same yeast cells on glycerol instead of glucose (Fig. S6 bottom row). Glycerol is a non-fermentative substrate and hence cells grown on glycerol can only obtain energy from respiration and oxidative phosphorylation. It is evident that cells grown on glycerol (which do not exhibit glycolytic oscillations) develop many more mitochondria than the cells with oscillating glycolysis. Thus, we conclude that while a minor fraction of the ACDAN signal undoubtedly stems from the mitochondria (1–2% of the cytoplasmic volume), most of the ACDAN signal comes from other parts of the cytoplasm and oscillations in all cytoplasmic compartments (except for the central vacuole) seems to be coupled. Next, we ensured that the ACDAN signal was not contaminated by NADH fluorescence. Fig. S7 shows that for two photon excitation of unlabelled controls cells excited at either 780 nm or at 810 nm, the NADH signal was not distinguishable from the noise at the laser intensity used. This finding is supported by the observation that ACDAN and NADH oscillate in antiphase (see above), excluding the possibility that NADH contributes to the ACDAN signal.

### Evidence of a narrow region of ACDAN GP where oscillations may occur

**Figure 2:**
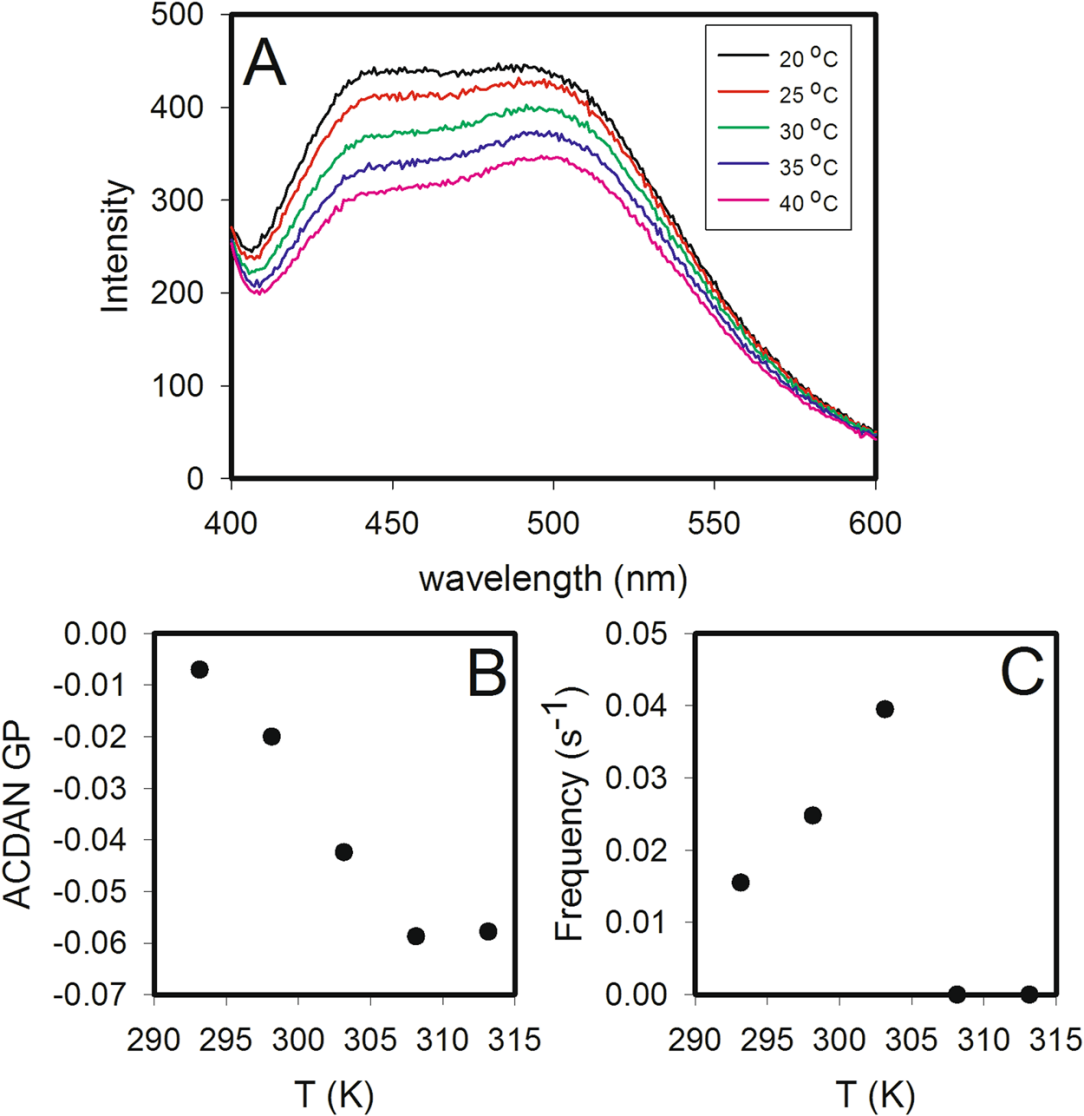
Fluorescence spectra of resting yeast cells incubated with ACDAN and plots of GP and oscillation frequency versus temperature. (**A**) Fluorescence spectra of 10% (w/v) *S. cerevisiae* BY4743 wild type strain stained with 10 μM ACDAN and measured at temperatures of 20, 25, 30, 35 and 40 °C. (**B**) Plot of GP values, calculated using equation (S1) and data from the spectra in A, against the measurement temperature. (**C**) Plot of oscillation frequency against the measurement temperature. Cells were grown at 30 °C.

**Figure 3:**
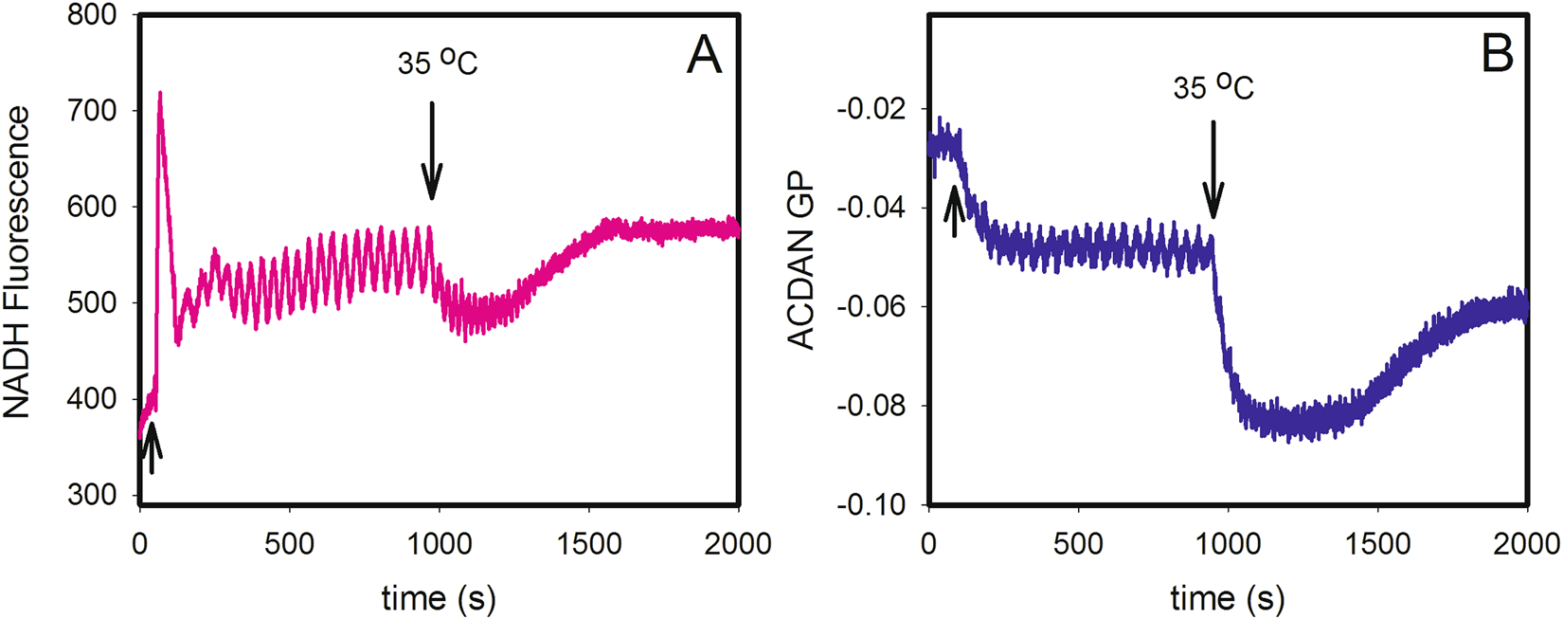
A temperature change shifts the ACDAN GP out of the oscillating region and destroys the oscillations. Oscillations in (**A**) NADH autofluorescence and (**B**) ACDAN GP in BY4743 wild type cells grown at 30 °C. The temperature was initially 25 °C. Oscillations were induced by the addition of first 30 mM glucose (arrow) and 60 s later 5 mM KCN as indicated by the arrows. At approximately 900 s, the temperature was changed from 25 °C to 35 °C.

We then decided to explore the effect of temperature on the observed oscillations. Figure 2A shows the fluorescence spectra of starved yeast cells grown at 30 °C incubated with 10 μM ACDAN for one hour at room temperature and measured at temperatures of 20, 25, 30, 35 and 40 °C. Included in the figure are plots of the ACDAN GP values versus temperature (Fig. 2B) and the frequency of the glycolytic oscillations (computed using FFT) versus temperature (Fig. 2C). The corresponding time series of NADH at the four different temperatures are shown in Fig. S8. We conducted similar experiments in cells grown at 25 °C and 35 °C, and the data are included in Figs S9–S12. Table S1 lists the resulting ACDAN GP values and oscillation frequencies at the different measurement temperatures. An oscillation frequency of 0 indicates that the FFT algorithm could not detect oscillations above the baseline noise. It is noteworthy that in cells grown at 35 °C, oscillations could only be detected at the lowest measured temperature (20), whereas for cells grown at 25 °C and 30 °C, oscillations occurred at the lower four and three temperatures, respectively. At the same time, it is evident that the ACDAN GP values showed a general decrease with increasing growth temperature. Thus, from the data in Table S1, it seems that oscillations only occur within a certain region of ACDAN GP values (approximately between 0.0 and −0.06). This was further substantiated by the experiment shown in Fig. 3, where oscillations were induced in cells grown at 30 °C and then after approximately 900 s the temperature was changed from 25 °C to 35 °C. We note that the increase in temperature induced first an increase in frequency accompanied by a decrease in amplitude, and thereafter the oscillations stopped. At the same time, the ACDAN GP dropped to a negative value (−0.08) outside the region where oscillations were observed.

### The critical ACDAN GP region is flanked by two supercritical Hopf bifurcations

**Figure 4:**
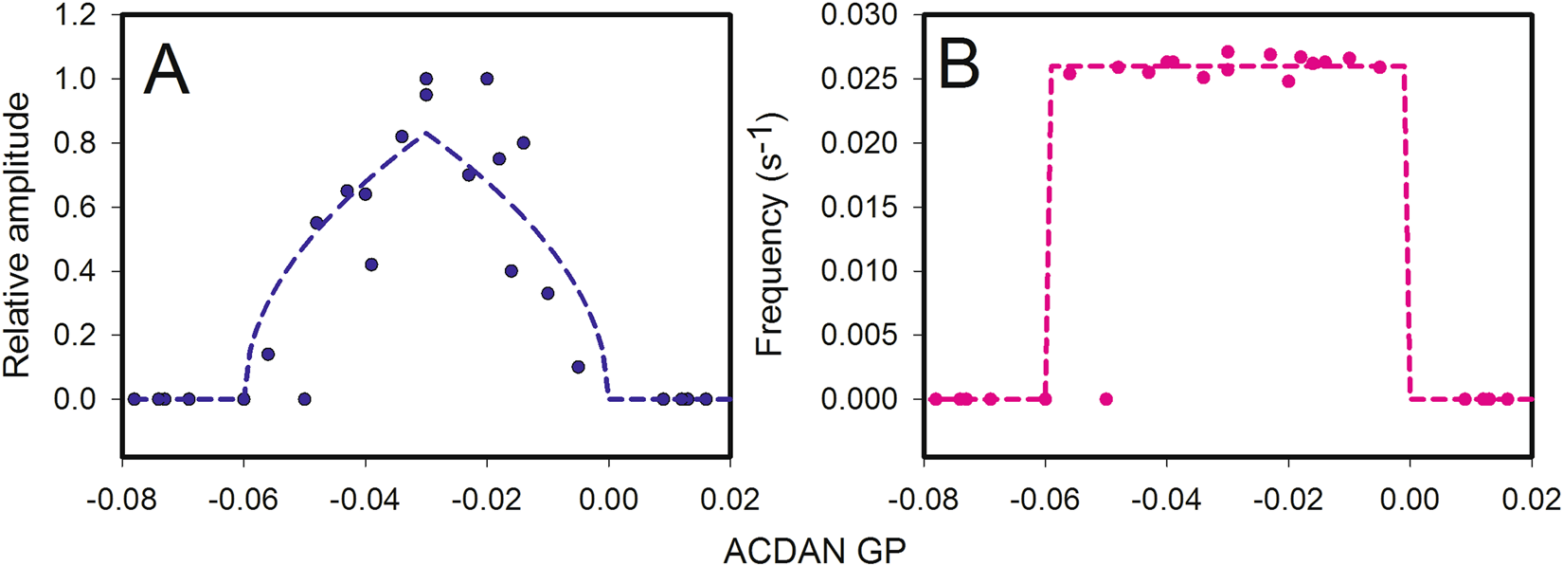
Plots of the glycolytic (NADH) oscillation amplitude (**A**) and the frequency (**B**) against the ACDAN GP of the wild type (*S. cerevisiae* BY4743) and 24 strains with isogenic mutations of various proteins listed in Table S2. All strains were grown at 30 °C and measured at 25 °C. The oscillation amplitude is scaled relative to the amplitude of NADH oscillations of the wild-type strain (*S. cerevisiae* BY4743).

To establish whether there is a region of ACDAN GP values in which glycolytic oscillations can arise, we looked for correlations between oscillations and ACDAN GP values in the BY4743 wild type and 24 isogenic strains with mutations in various enzymes affecting glycolysis. These included mitochondrial electron transport, ATP synthesis, vacuolar ATPases and proteins involved in actin polymerization and microtubule formation. The strains are listed in Table S2. All strains were grown at 30 °C and measured at 25 °C. The results are shown as plots of relative oscillation amplitude (Fig. 4A) and frequency (Fig. 4B) against ACDAN GP. The results obtained for the wild type strain grown at 25 °C and 35 °C are not included in the plots. The data in Fig. 4 support the idea that oscillations are only found within a certain region of ACDAN GP values, with the exception of the data corresponding to a GP value of −0.05, which belonged to a non-oscillating strain (YGR240c) that has a mutation in Phosphofructokinase 1. However, it is worth noting that when grown in a different laboratory, this strain shows NADH oscillations with a reduced amplitude^43^. When grown in our laboratory, this strain was previously shown to have a much-reduced glycolytic flux^44^, which could explain why no oscillations were observed. For the other 24 strains, it was not possible to establish a correlation between the glycolytic flux and the amplitude or frequency of the oscillations. A plot of the ACDAN GP versus the rate of ethanol production, which is a crude estimate of the glycolytic flux, measured in the 25 strains showed a random scatter of points (Fig. S13).

The data in Fig. 4 could be modelled by the following equations^45^:

1 $$\begin{array}{c}\frac{{\rm{d}}x}{{\rm{d}}t}=\mu x-\omega y-({x}^{2}+{y}^{2})x\\ \frac{{\rm{d}}y}{{\rm{d}}t}=\omega x+\mu y-({x}^{2}+{y}^{2})y\end{array}$$

where $\mu =k({\rm{GP}}-{{\rm{GP}}}_{0})$. GP is the ACDAN GP calculated according to equation (S1), GP_0_ is a constant determining the threshold for onset of the oscillations, *k* is a constant that can be fitted to the experimental data, and *ω* is the angular frequency of the oscillations. For *μ* > 0, this equation has the asymptotic solution:

2 $$\begin{array}{c}x=r\,\cos \,(\omega t)\\ y=r\,\sin \,(\omega t)\end{array}$$

where $r=\sqrt{\mu }$. For *μ* < 0, the solution to equation 1 is a damped oscillation. Thus, at *μ* = 0, we have a supercritical Hopf bifurcation. The amplitude and frequency data in Fig. 4 indicate two supercritical Hopf bifurcations, one around an ACDAN GP value of 0 and another at a value of −0.06. These two bifurcations were fitted by equation 1 using *k* = 22.68, *ω* = 0.157 *s* ^−1^ and GP_0_ = −0.06 for the bifurcation to the left and *k* = −22.68, *ω* = 0.157 *s* ^−1^ and GP_0_ = 0 for the bifurcation to the right. The fits are indicated by the dashed curves in Fig. 4. Note that although the relative oscillation amplitudes have values in the region from 0 to 1, the frequency is essentially constant within the GP region from −0.06 to 0. It is well known that in intact yeast cells, the frequency of glycolytic oscillations is relatively constant and affected only slightly by a few factors (other than temperature) such as hexose transport^46^, cell density^47^, and D_2_O^16^.

**Figure 5:**
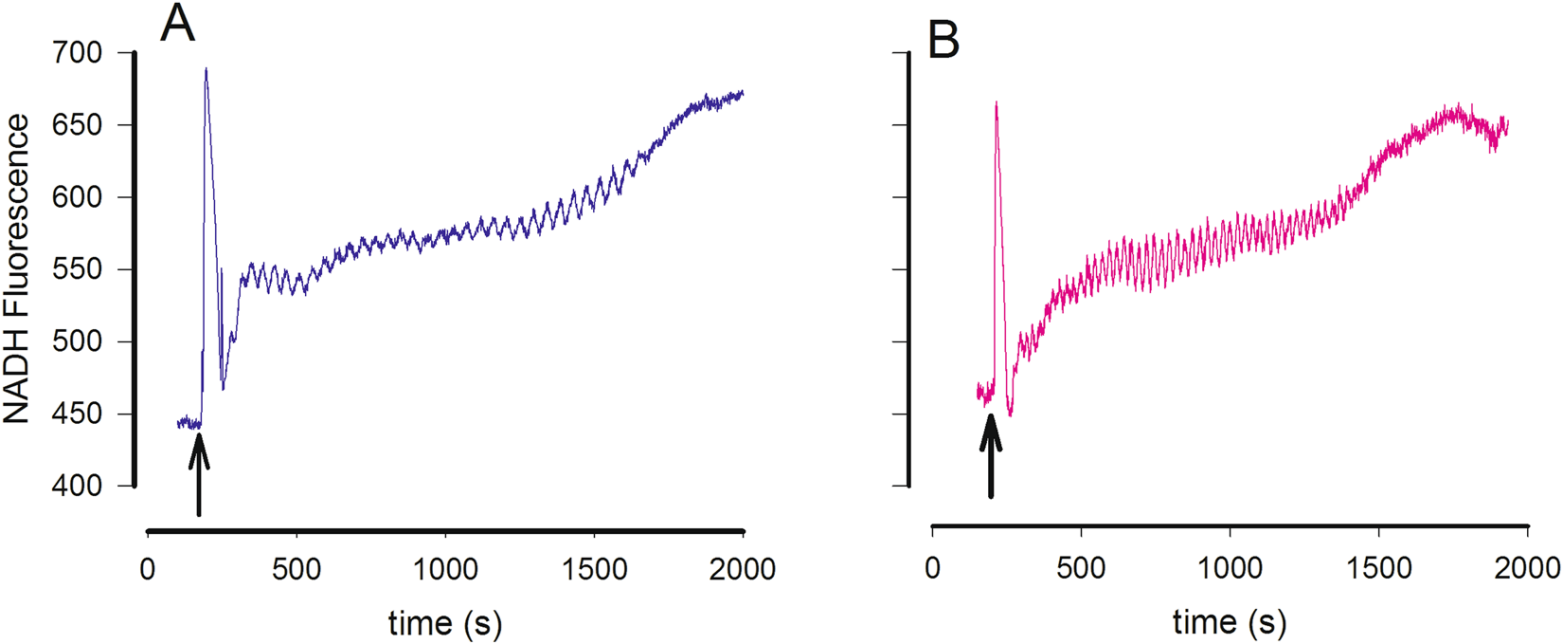
Oscillations of NADH following addition of glucose and KCN to a 10% (w/v) suspension of YIL034c mutant cells at (**A**) 25 °C and (**B**) 30 °C. Yeast cells were grown at 30 °C. Oscillations were induced by addition of first 30 mM glucose (arrow) and 60 s later 5 mM KCN.

To further investigate the correlation between ACDAN GP and NADH oscillation amplitude, we selected the mutant YIL034c, which has an ACDAN GP of −0.01 at 25 °C. This GP value is close to one of the two bifurcation points shown in Fig. 4. In this mutant strain, the amplitude of NADH oscillations was approximately 33% of the amplitude in the wild type at 25 °C. Oscillations in NADH at this temperature are shown in Fig. 5A. Upon raising the measurement temperature to 30 °C, we observed a decrease in the ACDAN GP to −0.027. According to the data shown in Fig. 4 the amplitude of the oscillations of NADH should increase at this GP value. Figure 5B shows the oscillations of NADH in the mutant at 30 °C. Indeed, the amplitude of the oscillations increased at this temperature as predicted by the data in Fig. 4.

### Inhibitors of ATP synthesis abolish the oscillations and raise ACDAN GP

**Figure 6:**
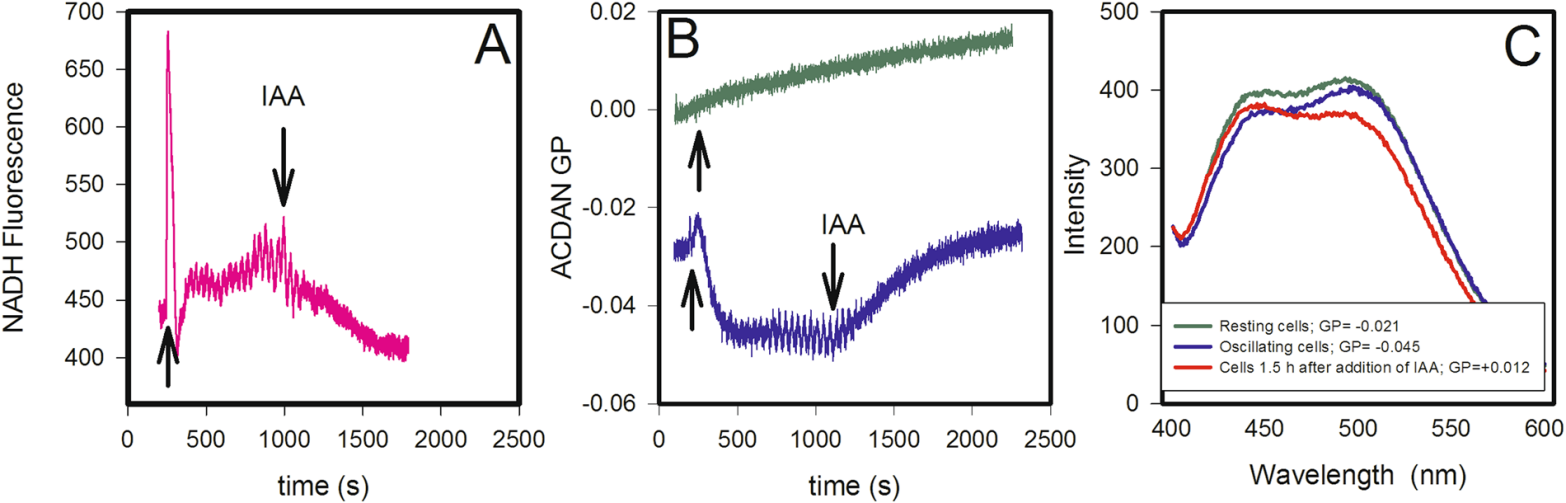
Iodoacetate eliminates oscillations and induces an increase in ACDAN GP. Oscillations in NADH (**A**) and ACDAN GP (blue curve in **B**) following addition of glucose and KCN to a 10% (w/v) suspension of BY4743 WT cells. At approximately 1100 s, 20 mM iodoacetate (IAA) was added to the suspension. The green curve in B represents cells that were incubated with ACDAN and iodoacetate for 1 h before glucose and KCN were added to the suspension. The ACDAN spectra in (**C**) represent resting cells before addition of glucose and KCN (green), oscillating cells (blue) and cells inhibited with iodoacetate, recorded 1.5 h after the addition of the inhibitor (red curve) The corresponding ACDAN GP values are listed in the box. Yeasts were grown at 30 °C. Oscillations were induced by addition of first 30 mM glucose and 60 s later 5 mM KCN. The measurement temperature was 25 °C.

We also tested the effect of inhibitors of glycolysis - and hence ATP synthesis - on ACDAN GP and NADH oscillations (Fig. 6). Here, we induced oscillations by treating cells with glucose and KCN and then adding the inhibitor iodoacetate, which blocks the activity of glyceraldehyde-3-phosphate dehydrogenase^48^. This brings the oscillations to a complete halt and induces an increase in ACDAN GP (blue curve in Fig. 6B). Approximately 90 min after the addition of iodoacetate, the ACDAN GP had increased to +0.012 (Fig. 6C red spectrum), i.e., outside the critical GP range where oscillations are observed (Fig. 4). Furthermore, incubating the yeast cells with iodoacetate for one hour before adding glucose and KCN brought the ACDAN GP to a positive initial value, and oscillations were no longer induced by the addition of glucose and KCN (green curve in Fig. 6B). 2-Deoxyglucose had a similar effect as iodoacetate, i.e., after the addition of 20 mM 2-deoxyglucose, the oscillations stopped, and the ACDAN GP increased toward positive values (Fig. S14). 2-Deoxyglucose is phosphorylated in a reaction catalysed by hexokinase^49^. However, the deoxyglucose-6-phosphate that is formed cannot be metabolized further, and hence, ATP production downstream in the pathway is inhibited, resulting in a decrease in glucose consumption and an accumulation of intracellular glucose^42,49^.

## Discussion

In a previous publication we presented evidence in *S. cerevisiae* of strong coupling between metabolic (glycolytic) oscillations and the dipolar relaxation dynamics of water^16^. Here, we extend these studies to include a more in-depth investigation, using the fluorescent probe ACDAN, of the influence of intracellular water dynamics in resting cells on glycolysis oscillation. As indicated in the Introduction, ACDAN is sensitive to the extent of dipolar relaxation induced by water in the probe milieu^16^. The results obtained using this method indicate that the physical state of water may be an important parameter in the occurrence of glycolytic oscillations. This is the first demonstration that an oscillating metabolic process is affected by a cell-wide intensive physical property, namely, the physical state of water. We hypothesize that the physical state of water, in terms of seldom-studied intensive variables such as viscosity, permittivity, and density, may influence many other metabolic and signalling processes.

As stated in the Introduction, there is still some controversy as to the state of water in intact cells. Some reports suggest that most intracellular water is in a fluid state^31^, whereas other reports claim that it is in a gel-like^28^ or polarized^20^ state. The results of our experiments on oscillations in glycolysis and water dynamics are consistent with the latter view. It is worth noting that the oscillations of ACDAN fluorescence can be measured in most parts of the cytoplasm, except for the central vacuole. It is also noteworthy that oscillations have been detected in the BZ reaction in viscous bulk solutions. In this case, oscillations in volume^32^ and viscosity^35^ were observed. The latter were shown to correlate with reversible aggregation of polymers, accompanied by oscillations in redox potential. Therefore, these oscillations may also involve oscillations in water dynamics.

The participation of water in the mechanism of glycolytic oscillations was further corroborated by the effect of D_2_O, which, due to its larger mass, increases the period of the oscillations^16^. It was noted that D_2_O only influences the frequency of the oscillations, not their amplitude. The effect of D_2_O is therefore different from the effect of a change in temperature, which affects both the frequency and the amplitude of oscillations (Figs S8, S10 and S12). The observation that the transition from steady-state to oscillation can be linked to changes in ACDAN GP in resting cells through supercritical Hopf bifurcations represents a strong indication that the dynamics of water, as measured by the GP, directly or indirectly, play an important role in the underlying mechanism of the oscillations. Similar supercritical Hopf bifurcations have been demonstrated for the dependence of glycolytic oscillations on the concentration of KCN^11^ and cell density^47^, both of which are critical factors in the generation of oscillations. It is interesting to note that the only factor which seems to link the occurrence of oscillatory behaviour in 25 different yeast strains is ACDAN GP. An explanation for how ACDAN GP and oscillating glycolysis are linked mechanistically can be provided by the Association-Induction hypothesis (A-I hypothesis) developed by G. Ling^20^. Briefly, this hypothesis proposes that the association of some central metabolites (e.g., ATP) with fibrillar proteins (i.e., those forming the cytoskeleton) causes conformational changes. In turn, these modulate the binding affinity of particular ions for proteins and the patterns of hydrogen bonding to water (as described by the polarized-oriented multilayer theory of cellular water^20,50^) and, hence, the partition coefficients of numerous molecular actors. We believe that the A-I hypothesis is relevant in light of the observed relationship between ATP and the oscillations of intracellular water dynamics. First, we found in a previous study, and confirmed here (see Fig. S3), that during oscillatory glycolysis, oscillations of the DAN probes are strictly in phase with the ATP oscillations^16,51^. Second, as reported in this paper, the experiments with iodoacetate and 2-deoxyglucose, which alter the normal production of ATP in the cell, inhibit the oscillations measured in NADH and ACDAN GP and push the ACDAN GP out of the oscillatory GP region.

**Figure 7:**
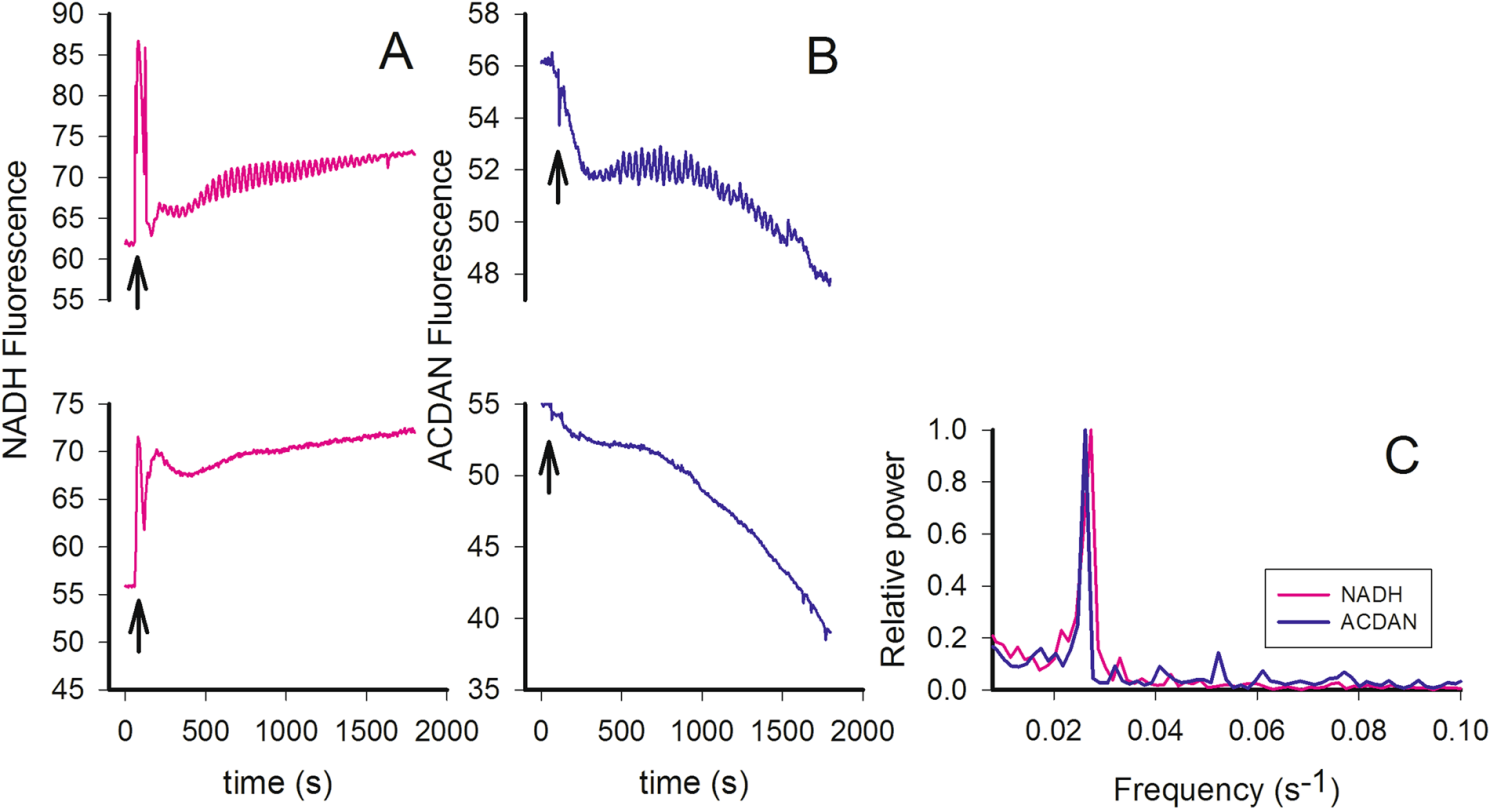
Latrunculin B obliterates oscillations in NADH and ACDAN. (**A**) NADH fluorescence. Top: Control incubated with 1% DMSO (v/v) shows strong oscillations following addition of 30 mM glucose and 60 s later 5 mM KCN. Bottom: After 3 hours of incubation at 30 °C with 600 μM latrunculin B. (**B**) ACDAN fluorescence. Top: Control was further incubated with 10 μM ACDAN. Bottom: Incubation with latrunculin B (as above). (**C**) FFT analysis of the oscillations shown in (**A**) and (**B**). The cells were grown at 30 °C, and the measurements were done at 25 °C.

To further test the adequacy of the A-I hypothesis to interpret our experimental results, i.e., to explore the potential participation of cytoskeleton proteins on the dynamic coupling observed between intracellular water and the metabolic process, we performed additional experiments using latrunculin B (see Fig. 7). Once incorporated in the cell, latrunculin B binds to actin monomers, preventing protein polymerization and therefore compromising the integrity of the actin network. Following the A-I hypothesis, we further hypothesize that the inability of actin to form fibrillary structures should affect the amount of polarized water in the cytosol. The results in Fig. 7 show that when actin polymerization was inhibited by latrunculin, oscillations of both NADH and ACDAN fluorescence are obliterated. Remarkably, upon latrunculin treatment, the GP value, which is indicative of the global extent of relaxation of intracellular water, moves out of the critical GP range with respect to the control (from a GP value of −0.02 for the oscillating control to a GP of 0.05 for the latrunculin-treated sample). In terms of the measured extent of water dipolar relaxation, the experiments show a similar behaviour to cells treated with iodoacetate and 2-deoxyglucose (Figs 6 and S14, respectively), i.e., the GP value increases. Together these experiments suggest that not only ATP but also the integrity of the cytoskeleton, are important players in the generation of glycolytic oscillations. An economical and coherent interpretation of the ensemble of experimental findings observed during glycolytic oscillations places the dynamic state of cytoplasmic water at the centre of the stage. This state may be modulated via reversible interactions of ATP with cytoskeleton proteins, which cyclically polarize and depolarize water via inductive effects, as proposed in the A-I hypothesis^20^. The oscillatory state requires an uncompromised cytoskeleton as well as the generation of “optimal” levels of ATP. Cells displaying glycolytic oscillations may be characterized by a low entropy state, which allows cell function under fermentation. Importantly, the fact that there is a narrow range of GP values (or initial extent of intracellular water dynamics, Fig. 4) limiting the occurrence of glycolytic oscillations may indicate that a structural balance is required for the cell to operate this way. In this context, it is important to emphasize that GP function was the only parameter found to correlate with oscillations in the 25 yeast strains; there was no correlation between oscillatory behaviour and other relevant parameters such as glycolytic flux (Fig. S13). This fact, along with the observations that i) most mutations in proteins directly associated with glycolysis do not inhibit oscillations^43,44^ and ii) several mutations in proteins that are usually not considered in the context of glycolysis do inhibit the oscillations, makes a mechanism based on local mass action kinetics as the sole explanation very unlikely. Instead, these observations suggest that glycolysis, at least in part, is controlled by a cell-wide property (i.e., the physical state of water), which through the A-I mechanism can transfer a response to the relevant glycolytic enzymes. The fact that, e.g., enzymatic activity^52,53^ or redox potential^32–35^ can be affected by the physical state of water makes it plausible that the dynamic behaviour of glycolysis is also affected.

The increase in GP values caused by energy depletion (2-deoxyglucose and iodoacetate) or disturbance of the cytoskeleton proteins (latrunculin B) suggests that the overall relaxation time of the intracellular water decreases in the cytoplasm. These findings plus our previous observation that the oscillation in water dynamics is a scale independent phenomenon^16^, support the idea that the global organization of the cytoplasm is affected by these perturbations. In a recent article, Munder *et al*.^29^ studied the effect of energy depletion on the mobility of particles in the cytosol of budding yeast. These authors concluded that energy depletion led to loss of ATP and acidification of the intracellular space, inducing a phase transition in the cytosol from a fluid to a solid-like state^29^. The authors concluded that this phenomenon involves a pH-dependent interaction of cytosolic proteins forming large clumps or filamentous structures that result in the cytoplasm becoming stiffer. Munder *et al*. also found that energy depletion causes a reduction in cell volume and suggested that it may be due to dehydration of the intracellular environment^29^. Additionally, the authors also observed that under conditions where ATP levels are not compromised, latrunculin treatment of the cells shows a similar, but more moderate, effect^29^. Importantly, according to our measurements, the extent of intracellular water relaxation decreased under similar conditions (Fig. 7).

In a similar study, Joyner *et al*.^30^ found that glucose depletion led to decreased molecular mobility in both the cytoplasm (mRNPs) and the nucleus (chromatin). In contrast to Munder *et al*.^29^ they conclude, however, that the effect is not pH driven, but caused by an increased macromolecular crowding following the decrease in cell volume, leading to an increased ‘microscopic’ viscosity. Interestingly, we also observed that glucose depletion results in an increase in the GP value (that is, a decrease in the extent of water relaxation) compared to that observed during glycolytic oscillation (Fig. S1) and a decrease in intracellular pH. By extending the starvation to very long times, i.e., more than 10 hours, the measured GP values move out of the observed critical GP range, and oscillations can no longer be induced (Fig. S15).

It is worth noting that the ACDAN GP value decreases at increasing temperatures affecting, particularly at higher temperatures, the occurrence of glycolytic oscillations (Table S1). The observed decrease in GP is consistent with an increase in water dynamics induced by increasing the temperature, which gradually moves the extent of relaxation to one of the edges or outside of the critical GP range observed in Fig. 4 (as shown in Figs 3 and 5). This effect of temperature on GP was extensively demonstrated for LAURDAN inserted in lipid bilayers when thermotropic phase transitions are induced^54^. We hypothesize that the observed temperature effect is consistent with the idea of a system transitioning from an optimum state to a stressed one, i.e., the extra thermal energy (k_B_T) provided to the system by increasing temperature may disturb the overall organization of the cytoplasm.

Finally, to further support the claim that our results may be explained by the A-I hypothesis, we constructed a model using the Yang-Ling isotherm for cooperative adsorption to a polymer^55,56^. The model places phosphofructokinase at the centre of the metabolic oscillations and assumes that the ATP produced in glycolysis is adsorbed to intracellular proteins (e.g., actin) and this adsorption induces structuration of the intracellular water to a more polarized state (*p*). The model is presented in the Supplementary Information, and two simulations are presented in Figs S16 and S17. Figure S16 shows that intracellular ATP and the polarization of water (*p*) oscillate in phase, as we observed for ATP and ACDAN GP (see Fig. S3), and Fig. S17 shows that there is a narrow region of steady state values of *p*, which allow for oscillations. This region is flanked by two supercritical Hopf bifurcations similar to those in Fig. 4A. Interestingly, this model may provide an explanation for why oscillations appear cell-wide and most cellular regions seem to oscillate in phase. Given restrictions on diffusion in crowded environments, a mechano-chemical coupling via structured or polarized water, which is controlled by adsorption of ATP to proteins and other intracellular surfaces, can account for an almost instantaneous spatiotemporal coupling of the oscillations. Conversely, cellular regions where glycolytic oscillations are absent may show oscillations in the polarization of water coupled to the polarization of water in the regions exhibiting glycolytic oscillations, but with a slight phase shift (Figure S18). For example, one would not expect to observe metabolic oscillations in intracellular lipid droplets. To test this hypothesis, we simultaneously measured ACDAN fluorescence and the fluorescence of Nile red, which partitions into intracellular lipid domains, e.g., lipid droplets^57^. Nile red fluorescence also responds to the polarity of water^58^. Here, we did indeed observe oscillations of both probes (see Fig. S19), but with a slight phase shift, indicating that the Nile red fluorescence is slightly delayed compared to the ACDAN signal, as predicted by the proposed model. To demonstrate that our results with the simple Yang-Ling model are general and not limited to this particular model, we implemented the Yang-Ling approach on a full-scale model of glycolysis adapted from the model by Hald and Sørensen^12^. Figure S20 illustrates the network structure of the model, and Fig. S21 shows a simulation as phase plots of the polarization of water (*p*) versus [ATP] and *p* versus [NADH]. Note the similarity of these plots to the experimental plots in Fig. S3C and D. As in the experiments, the model shows that *p* oscillates 180° out of phase with NADH, whereas *p* and ATP oscillate in phase. This result also puts intracelullar water dynamics at the center of the stage, giving this emergent property an important role in optimizing cell function under stressful conditions.

## Methods

### Chemicals

6-acetyl-2-dimethylaminonaphthalene (ACDAN) was purchased from Santa Cruz Biotechnology Inc. (Dallas, TX). MitoTracker Red and CFDA-SE ester were from Thermo-Fisher Scientific (MA, USA). All other chemicals were from Sigma-Aldrich (Munich, Germany).

### Yeast strains and growth

The yeast strains used in this study are listed in Table S2. The strains, all obtained from EUROSCARF (Frankfurtam Main, Germany), were grown as described previously^44^. Before use, the cells were washed and resuspended in 100 mM potassium phosphate to a cell density of 10% (w/v) and starved for 3 h on a rotary shaker at 30 °C.

### Cuvette measurements

Measurements of NADH and ACDAN fluorescence were made in a QE65000 spectrometer (Ocean Optics, Dunedin, FL) fitted with a temperature-controlled cuvette holder (Quantum Northwest, Liberty Lake, WA, USA). The temperature of the sample was maintained at 25 ± 0.01 °C unless otherwise stated. Light was supplied by a CoolLED pE4000 illumination system (CoolLED, Andover, UK). The optical fibre from the illumination system was mounted perpendicular to the emission beam. NADH was excited at 365 (10 nm width at half height), and fluorescence emission was measured as the average intensity in the wavelength range 435 nm-450. ACDAN was excited at 365 nm (10 nm width at half height), and fluorescence emission was measured as the average intensity in the wavelength range 435 nm–445 nm and 485 nm–495 nm. Spectra of ACDAN emission were measured at a 1 nm resolution. All emissions recorded represent the average of 5 repeats at 100 ms intervals. Some experiments measuring NADH and ACDAN fluorescence were also conducted in an Edinburgh FL920 spectrometer fitted with the same temperature-controlled holder (Quantum Northwest) as mentioned above. In these cases, NADH was excited at 366 nm (slit 3 nm) and emission was measured at 450 nm (slit 10 nm). ACDAN was excited at 370 nm, and ACDAN emission spectra were measured with a slit of 1 nm.

### Microscopy

Fluorescence images of ACDAN were obtained on an inverted multiphoton excitation fluorescence microscope (Zeiss LSM 510 META NLO, Carl Zeiss, Jena, Germany) equipped with a Ti:Sa MaiTai XF-W2S laser (Broadband Mai Tai with 10 W Millennia pump laser with a tuneable excitation range of 710–980 nm, Spectra Physics, Mountain View, CA). The fluorescence signal was collected through either a 63X water objective, NA 1.2 or a 63X oil objective, NA 1.4. For two-photon excitation, the signal was split to two detectors (H7422 PMT, Hammamatsu, Denmark) by a 460 nm long-pass dichroic mirror and then filtered by a 520 ± 17.5 nm, 462 ± 24 nm or a 438 ± 12 nm bandpass filter (all from AHF Analysen Technik AG, Tübingen, Germany). Acquisition of the two-photon excitation fluorescence image was performed by adding 300 μl of ACDAN-labelled resting yeast cells at a density of 10% by weight (suspended in 100 mM potassium phosphate buffer, pH 6.8) to an 8-well plastic chamber (Lab-tek Brand Products, Naperville, IL). The chamber containing the sample was placed in the microscope, and the images were acquired a few minutes later to allow the cells to sink to the bottom of the well. For measurement of NADH signal, an additional 30 mM glucose was added to the buffer. Image analysis was done using either a MATLAB script or ImageJ.

### Staining of cells with ACDAN, CFDA-SE and MitoTracker Red

Yeast cells (10% w/v) in 100 mM potassium phosphate buffer, pH 6.8, were incubated at 30 °C with 5 μM–20 μM ACDAN, 4.5 μM CFDA-SE ester, 1 μM MitoTracker Red or a combination of the three for 1 h, washed twice and finally resuspended in the same buffer.

### Measurement of oscillations

Yeast cells at 10% (w/v) in 100 mM phosphate buffer, pH 6.8, were added to a 2 mL cuvette mounted in the fluorometer under stirring conditions. Oscillations were induced by first adding 30 mM glucose and 60 s later 5 KCN to the suspension.

### Inhibiting oscillations

Inhibitors of glycolysis (iodoacetate or 2-deoxyglucose) were either added directly to cell suspensions after oscillations were induced by addition of glucose and KCN, or the cells were incubated with the inhibitor at 30 °C for 0.5 h–1 h prior to the addition of glucose and KCN. For inhibition with latrunculin B, the cells were incubated with the inhibitor at 30 °C for 3 h before oscillations were induced by glucose and KCN.

### Measurements of glycolytic flux

Glycolytic flux was measured either as the rate of production of ethanol using membrane-inlet mass spectrometry or using the NADH time series^8,59^. For the latter, it was shown previously that the flux is inversely proportional to the time from addition of glucose and KCN until the NADH fluorescence reaches a maximum^59^. Through calibration curves, it was established that the measured ethanol accounts for more than 80% of the glucose added.

### Determination of amplitude and frequency of oscillations

The amplitude of NADH oscillations was measured as the average of the 20 largest excursions of a time series. The frequency was measured using FFT using either MATLAB or the Berkeley Madonna algorithm.

### Data availability

The data that support the findings of this study are available from the corresponding author upon reasonable request.

## Electronic supplementary material

Supporting Information
