# Supplementary material for: The dynamics of intracellular water constrains glycolytic oscillations in Saccharomyces cerevisiae
Source: Sci Rep. 2017 Nov 24;7:16250. doi: 10.1038/s41598-017-16442-x (PMC5701229; doi:10.1038/s41598-017-16442-x)
Supplement: Supplementary file 1 — Supporting Information [file 41598_2017_16442_MOESM1_ESM.pdf]

Supplementary Information

The dynamics of intracellular water constrains glycolytic oscillations in *Saccharomyces cerevisiae*

Henrik S. Thoke[1,2], Sigmundur Thorsteinsson[2] Roberto P. Stock[1], Luis A. Bagatolli[1,3], and Lars F. Olsen[1,2,*]

[1]Center for Biomembrane Physics (MEMPHYS) and [2]Institute for Biochemistry and Molecular Biology, University of Southern Denmark, Campusvej 55, DK5230 Odense M, Denmark. [2]Yachay EP and Yachay Tech, Yachay City of Knowledge, 100650 Urcuquí-Imbabura, Ecuador

[*]Corresponding author: lfo@bmb.sdu.dk

1. Supporting Methods:

The generalized polarization (GP) function was originally introduced as an analytical method to quantitatively determine the relative amounts and temporal fluctuations of two distinct lipid phases when they coexist in a model membrane, for reviews see (1,2). This function was originally defined as:

$$GP = \frac{I_B - I_R}{I_B + I_R} \quad \text{(S1)}$$

where I_B and I_R are the measured fluorescence intensities under conditions in which a wavelength (or a band of wavelengths) B (for blue shifted) and R (for red shifted) are both observed using a given excitation wavelength. Being a weighted difference, the values of the GP must fall within -1 and 1; the lower the value the greater the extent of relaxation (or bathochromic shift of the spectrum). This definition is formally identical to the classical definition of fluorescence polarization, in which B and R represent two orthogonal orientations of the observation polarizers in the fluorimeter. The advantage of the GP function for the analysis of the spectral properties of the DAN probes is derived from the well-known properties of the classical polarization function, which contains information on the interconversion between two different "states" of the emitting dipole of the fluorophore. In the original studies, the LAURDAN GP was shown to distinguish between the extent of water relaxation in solid-ordered (s_o) and liquid-disordered (l_d) phases in phospholipid membranes (1,2). In the GP function as used here, the two states correspond to the unrelaxed and relaxed environments sensed by the probes. Our approach to the study of intracellular water dynamics in yeast therefore constitutes a generalization of the use of the GP function (3, 4). In this case, however, we explore fluctuations in water relaxation throughout the cell rather than in just membrane-associated water. The oscillations of the GP function in the cell (Figure S1C) yielding the measured changes in the intensity of emission (quantum yield; Figure S1B) of the probes at any given wavelength can be explained *only if solvent relaxation is the dominant mechanism.* In the classical definition of GP, B and R correspond to 440 and 490 nm, respectively, of the ACDAN fluorescence emission spectrum (1,2).

2. Modelling the coupling of glycolytic oscillations with water dynamics

In order to put our experimental results in to a more rigid theoretical framework we use the Association-Induction hypothesis proposed by GN Ling (5). This hypothesis builds on the assumptions (i) that the bulk of water and various solutes are adsorbed on cellular proteins, (ii) that this adsorption is synchronized as a result of interactions of neighbouring adsorption sites (cooperativity), and (iii) that the cooperative adsorptions are controlled by a smaller number of molecular species referred to as *cardinal adsorbents*, which exert their control by interacting with certain key sites (cardinal sites) on the same proteins (5).

Here we give a brief outline of a recently developed model for the coupling of glycolytic oscillations with intracellular water dynamics. The model is based on an earlier model of glycolysis (6) using the generally accepted view that *phosphofructokinase* is a key enzyme in controlling the pace of glycolysis and that the enzyme shows cooperativity with respect to binding of ATP and is activated by its product ADP (6). However, instead of using the classical mass-action based Michaelis-Menten (or for cooperative enzymes Monod-Wyman-Changeux) approach, we use Yang-Ling isotherms (5,7,8) for describing the transformation

of ATP to ADP and its coupling to the state of water, which is denoted p (for polarized). As opposed to the Monod-Wyman-Changeux and the related Koshland-Nemethy-Filmer models the Yang-Ling isotherm has a statistical-mechanical origin and is general.

The full description of the model will be presented elsewhere (manuscript in preparation), but briefly the equations are

$$\frac{d[ATP]}{dt} = v - v_{PFK} \qquad (S2)$$

$$\frac{d[ADP]}{dt} = v_{PFK} - k_1[ADP] \qquad (S3)$$

$$\frac{dp}{dt} = v_o - k_o p \qquad (S4)$$

where v_{PFK} is defined by:

$$v_{PFK} = V \cdot \left(\frac{[ADP]_{ads}}{F} \cdot R_1 + \left(1 - \frac{[ADP]_{ads}}{F}\right) \cdot R_2\right),$$

where $R_1 = \frac{1}{2}\left(1 + \frac{\frac{[ATP]}{K_C^{oo}} - 1}{\sqrt{\left(\frac{[ATP]}{K_C^{oo}} - 1\right)^2 + 4\frac{[ATP]}{K_C^{oo}}\exp(-\frac{\gamma_C}{RT})}}\right)$, $R_2 = \frac{1}{2}\left(1 + \frac{\frac{[ATP]}{K_O^{oo}} - 1}{\sqrt{\left(\frac{[ATP]}{K_O^{oo}} - 1\right)^2 + 4\frac{[ATP]}{K_O^{oo}}\exp(-\frac{\gamma_O}{RT})}}\right)$ and

$$\frac{[ADP]_{ads}}{F} = \frac{1}{2}\left(1 + \frac{\frac{[ADP]}{\kappa_C^{oo}} - 1}{\sqrt{\left(\frac{[ADP]}{\kappa_C^{oo}} - 1\right)^2 + 4\frac{[ADP]}{\kappa_C^{oo}}\exp(-\frac{\Gamma}{RT})}}\right)$$

Here V is a maximal activity of PFK, while $K_C^{oo}, K_O^{oo}, -\frac{\gamma_C}{2}$ and $-\frac{\gamma_O}{2}$ represent ATP dissociation constants for adsorption of ATP to sites on PFK and nearest-neighbour interaction energies under the control of ADP ($K_C^{oo}$and $-\frac{\gamma_C}{2}$) and in the absence of control ($K_O^{oo}$and $-\frac{\gamma_O}{2}$, in contrast to Ling (5,8) we use dissociation constants instead of association constants). κ_C^{oo} represents the dissociation constant for binding of ADP to a cardinal binding site while $-\frac{\Gamma}{2}$ is the nearest neighbour interaction energy for exchanging H_2O with ADP at this site. R is the gas constant, while T is the absolute temperature. F is the concentration of adsorption sites for ADP.

The symbol v in equation S2 represents a constant supply of ATP, while k_1 and k_o in equations S3 and S4 are simple first order rate constants. The rate v_o in equation S4 is given by the expression (5):

$$v_o = \frac{V_1}{2}\left(1 + \frac{\frac{[ATP]}{K_{c1}^{oo}} - 1}{\sqrt{\left(\frac{[ATP]}{K_{c1}^{oo}} - 1\right)^2 + 4\frac{[ATP]}{K_{c1}^{oo}}exp(-\frac{\gamma_{c1}}{RT})}}\right)$$

where V_1 is a maximum rate for the ATP-induced transition of water from a less to a more polarized state (p) and K_{c1}^{oo} and $-\frac{\gamma_{c1}}{2}$ represent the dissociation constant and the nearest neighbour interaction energy for binding of ATP to fibrillary proteins, e.g. actin.

We now assume that the maximum activity of PFK (V) and the dissociation constant for binding of ATP (K_c^{oo}) depend on the variable p, i.e. V(p) and $K_c^{oo}(p)$. It is well-documented that for enzymes in viscous solutions the maximum activity and the binding constants of substrates change with crowding (9,10). For simplicity we assume that *V* is inversely proportional to *p*, while $K_c^{oo}$is proportional to *p*. However, other relations between the two parameters and *p* will yield similar behaviour. Furthermore, it is quite possible that many of the other parameters in equations *S2-S3* may depend on *p*, but again this will only have qualitative effect on the behaviour of the model. In the current form of the model ATP, ADP , p and time t appear as dimensionless variables. The model was simulated using the Berkeley-Madonna software (Berkeley-Madonna, Berkeley, CA)

Simulations of the model are shown in Figs. S16 and S17. The data in Fig. S16 reveal that ATP and *p* oscillate in phase as revealed by the phase plot in Fig. S16C. Note that this phase plot is similar to that of ATP and ACDAN fluorescence (Fig. S3C). Furthermore, reducing either the maximum velocity V_1 or increasing the rate constant k_o will destroy the oscillations. This situation corresponds to that in Fig. 6 where the formation of actin filaments is inhibited by Latrunculin B. Changing the rate constant k_o will change the steady-state level of p, which again will affect the amplitude and the frequency of the oscillations. A plot of the relative amplitude of oscillations of ATP against the steady-state value of *p* reveals a double Hopf bifurcation (Fig. S17), similar to the experimental Hopf bifurcation shown in Fig. 4A. Finally, the model predicts that simple mechanical coupling of *p* in a region where glycolytic oscillations occur to the polarization of water (p_1), in a region where glycolytic oscillations are absent, would result in a slight phase shift in the oscillations of *p* and p_1 (Fig. S18), which is confirmed experimentally (Fig S19).

It should be emphasized that the variables in the model constitute a network (11), and hence one cannot say that metabolic oscillations drive oscillations in the polarization of water or the other way around. Furthermore, in such a network one cannot study the individual components in isolation, e.g. by assuming that situations may exist in which one variable shows oscillations while others do not.

To investigate if the results obtained with the simple model (equations S2-S4) are general we also implemented the Yang-Ling approach to the coupling of the polarity of water to glycolytic oscillations on a detailed model of glycolysis adapted from Hald and Sørensen (12). The model, which involves 24 reactions and 32 chemical species, is shown in Fig. S20 and Fig. S21 shows two phase plots of *p* versus [ATP] and *p* versus [NADH], respectively. We note that the first phase plot is similar to those in Fig. S3C and S16C, showing that *p* is in phase with [ATP] as observed experimentally and with our simple model. While the simple model (equations S2-S4) does not involve NADH it is interesting to compare phase plots of *p* versus [NADH] in the detailed model (Fig. S21B) with the corresponding plots of ACDAN versus NADH fluorescence (Fig. S3D). Both plots show that the oscillations of ACDAN GP are in antiphase with oscillations in NADH.

Table S1 Corresponding values of growth temperature, measurement temperature, ACDAN GP and oscillation frequency for the wild type BY4743 S. cerevisiae strain. A frequency of 0 s^{-1} means that no oscillations were obtained at the particular temperature.

Growth temp (°C)	Measurement temp (°C)	ACDAN GP	Frequency (s^{-1})
25	20	0.0002	0.0151
25	25	−0.0154	0.0250
25	30	−0.0362	0.0377
25	35	−0.0440	0.0552
25	40	−0.0574	0
30	20	−0.0007	0.0155
30	25	−0.0200	0.0248
30	30	−0.0424	0.0395
30	35	−0.0587	0
30	40	−0.0578	0
35	20	−0.0274	0.017
35	25	−0.0520	0
35	30	−0.0701	0
35	35	−0.0817	0
35	40	−0.0845	0

Table S1 Yeast strains used in this study

Yeast strain	Gene deletion	Genotype
BY4743	-	MATa/MATα.; his3Δ1/his3Δ1 ; leu2Δ0/leu2Δ0; met15Δ0\MET15; LYS2/lys2Δ0; ura3Δ0/ura3Δ0
YFR053c	Hxk1Δ	BY4743; MAT a/α; his3Δ1/his3Δ1; leu2Δ0/leu2Δ0; lys2Δ0/LYS2 ; MET15/met15Δ0; ura3Δ0/ura3Δ0; YFR053c::kanMX4/YFR053c::kanMX4
YGL253w	Hxk2Δ	BY4743; MAT a/α; his3Δ1/his3Δ1; leu2Δ0/leu2Δ0; lys2Δ0/LYS2 ; MET15/met15Δ0; ura3Δ0/ura3Δ0; YGL253w::kanMX4/YGL253w::kanMX4
YGR240c	Pfk1Δ	BY4743; MAT a/α; his3Δ1/his3Δ1; leu2Δ0/leu2Δ0; lys2Δ0/LYS2 ; MET15/met15Δ0; ura3Δ0/ura3Δ0; YGR2240c::kanMX4/YGR240c::kanMX4
YGR254w	Eno1Δ	BY4743; MAT a/α; his3Δ1/his3Δ1; leu2Δ0/leu2Δ0; lys2Δ0/LYS2 ; MET15/met15Δ0; ura3Δ0/ura3Δ0; GR254w::kanMX4/YGR254w::kanMX4
YBL099w	Atp1Δ	BY4743; MAT a/α; his3Δ1/his3Δ1; leu2Δ0/leu2Δ0; lys2Δ0/LYS2 ; MET15/met15Δ0; ura3Δ0/ura3Δ0; YBL099w::kanMX4/YBL099w::kanMX4
YKL016c	Atp7Δ	BY4743; MAT a/α; his3Δ1/his3Δ1; leu2Δ0/leu2Δ0; lys2Δ0/LYS2 ; MET15/met15Δ0; ura3Δ0/ura3Δ0; YKL016c::kanMX4/YKL016cc::kanMX4
YDR377w	Atp17Δ	BY4743; MAT a/α; his3Δ1/his3Δ1; leu2Δ0/leu2Δ0; lys2Δ0/LYS2 ; MET15/met15Δ0; ura3Δ0/ura3Δ0; DR377w::kanMX4/YDR377w::kanMX4
YMR056c	Aac1Δ	BY4743; MAT a/α; his3Δ1/his3Δ1; leu2Δ0/leu2Δ0; lys2Δ0/LYS2 ; MET15/met15Δ0; ura3Δ0/ura3Δ0; YMR056c::kanMX4/YMR956c::kanMX4
YHR051w	Cox6Δ	BY4743; MAT a/α; his3Δ1/his3Δ1; leu2Δ0/leu2Δ0; lys2Δ0/LYS2 ; MET15/met15Δ0; ura3Δ0/ura3Δ0; HR051w::kanMX4/YHR051w::kanMX4
YMR145c	Nde1Δ	BY4743; MAT a/α; his3Δ1/his3Δ1; leu2Δ0/leu2Δ0; lys2Δ0/LYS2 ; MET15/met15Δ0; ura3Δ0/ura3Δ0; YMR145c::kanMX4/YMR145c::kanMX4
YDL085w	Nde2Δ	BY4743; MAT a/α; his3Δ1/his3Δ1; leu2Δ0/leu2Δ0; lys2Δ0/LYS2 ; MET15/met15Δ0; ura3Δ0/ura3Δ0; YDL085w::kanMX4/YDL085w::kanMX4
YDL185w	Vma1Δ	BY4743; MAT a/α; his3Δ1/his3Δ1; leu2Δ0/leu2Δ0; lys2Δ0/LYS2 ; MET15/met15Δ0; ura3Δ0/ura3Δ0; YDL185w::kanMX4/YDL185w::kanMX4
YKL080w	Vma5Δ	BY4743; MAT a/α; his3Δ1/his3Δ1; leu2Δ0/leu2Δ0; lys2Δ0/LYS2 ; MET15/met15Δ0; ura3Δ0/ura3Δ0; YKL080w::kanMX4/YKL080w::kanMX4
YBR026c	Etr1Δ	BY4743; MAT a/α; his3Δ1/his3Δ1; leu2Δ0/leu2Δ0; lys2Δ0/LYS2 ; MET15/met15Δ0; ura3Δ0/ura3Δ0; YBR026c::kanMX4/YBR026c::kanMX4
YHR129c	Arp1Δ	BY4743; MAT a/α; his3Δ1/his3Δ1; leu2Δ0/leu2Δ0; lys2Δ0/LYS2 ; MET15/met15Δ0; ura3Δ0/ura3Δ0; YHR129c::kanMX4/YHR129c::kanMX4
YIL034c	Cap2Δ	BY4743; MAT a/α; his3Δ1/his3Δ1; leu2Δ0/leu2Δ0; lys2Δ0/LYS2 ; MET15/met15Δ0; ura3Δ0/ura3Δ0; YIL034c::kanMX4/YIL034c::kanMX4
YCR088w	Abp1Δ	BY4743; MAT a/α; his3Δ1/his3Δ1; leu2Δ0/leu2Δ0; lys2Δ0/LYS2 ; MET15/met15Δ0; ura3Δ0/ura3Δ0; YCR088w::kanMX4/YCR088w::kanMX4
YNL020c	Ark1Δ	BY4743; MAT a/α; his3Δ1/his3Δ1; leu2Δ0/leu2Δ0; lys2Δ0/LYS2 ; MET15/met15Δ0; ura3Δ0/ura3Δ0; YNL020c::kanMX4/YNL020c::kanMX4
YIL095w	Prk1Δ	BY4743; MAT a/α; his3Δ1/his3Δ1; leu2Δ0/leu2Δ0; lys2Δ0/LYS2 ; MET15/met15Δ0; ura3Δ0/ura3Δ0; YIL095w::kanMX4/YIL095w::kanMX4
YAL047c	Spc72Δ	BY4743; MAT a/α; his3Δ1/his3Δ1; leu2Δ0/leu2Δ0; lys2Δ0/LYS2 ; MET15/met15Δ0; ura3Δ0/ura3Δ0; YAL047c::kanMX4/YAL047c::kanMX4
YGL216w	Kip3Δ	BY4743; MAT a/α; his3Δ1/his3Δ1; leu2Δ0/leu2Δ0; lys2Δ0/LYS2 ; MET15/met15Δ0; ura3Δ0/ura3Δ0; YGL216w::kanMX4/YGL216w::kanMX4
YML124c	Tub3Δ	BY4743; MAT a/α; his3Δ1/his3Δ1; leu2Δ0/leu2Δ0; lys2Δ0/LYS2 ; MET15/met15Δ0; ura3Δ0/ura3Δ0; YML124c::kanMX4/YML124c::kanMX4
YLR200w	Gim1Δ	BY4743; MAT a/α; his3Δ1/his3Δ1; leu2Δ0/leu2Δ0; lys2Δ0/LYS2 ; MET15/met15Δ0; ura3Δ0/ura3Δ0; LR200w::kanMX4/YLR200ww::kanMX4
YEL003w	Gim4Δ	BY4743; MAT a/α; his3Δ1/his3Δ1; leu2Δ0/leu2Δ0; lys2Δ0/LYS2 ; MET15/met15Δ0; ura3Δ0/ura3Δ0; YEL003w::kanMX4/YEL003w::kanMX4

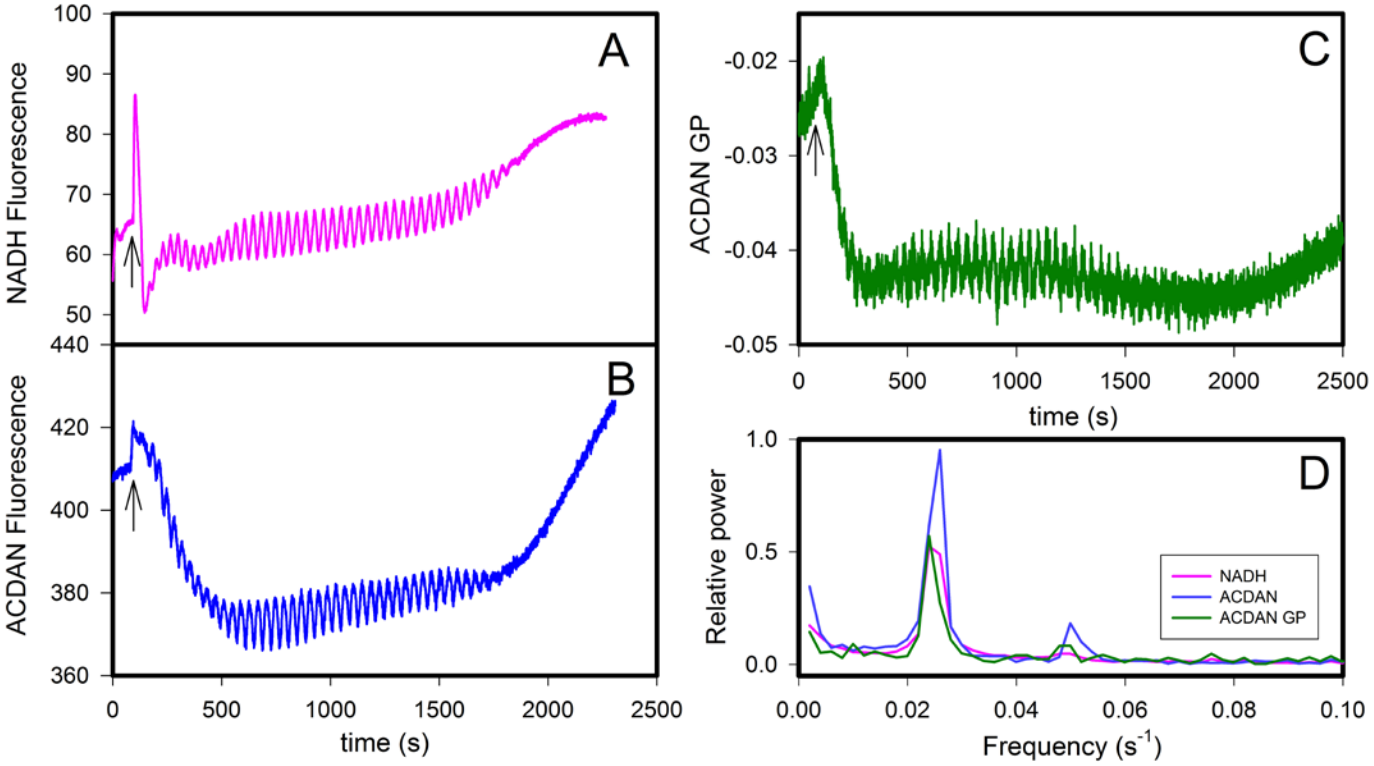

Figure S1. Oscillations of (A) NADH autofluorescence, (B) ACDAN fluorescence, and (C) ACDAN GP (calculated using equation (S1)) . (D) Power spectra of the time series indicated in A, B and C. *S. cerevisiae* BY4743 cells grown at 30 °C (10% w/v) were suspended at 25 °C in 100 mM potassium phosphate, pH 6.8. The cells in panels B and C were stained with 5 µM ACDAN for 1 h and then washed twice before suspension in potassium phosphate buffer. At the indicated arrows 30 mM glucose and (60 s later) 5 mM KCN were added to the cell suspension. Measurement temperature was 25 °C.

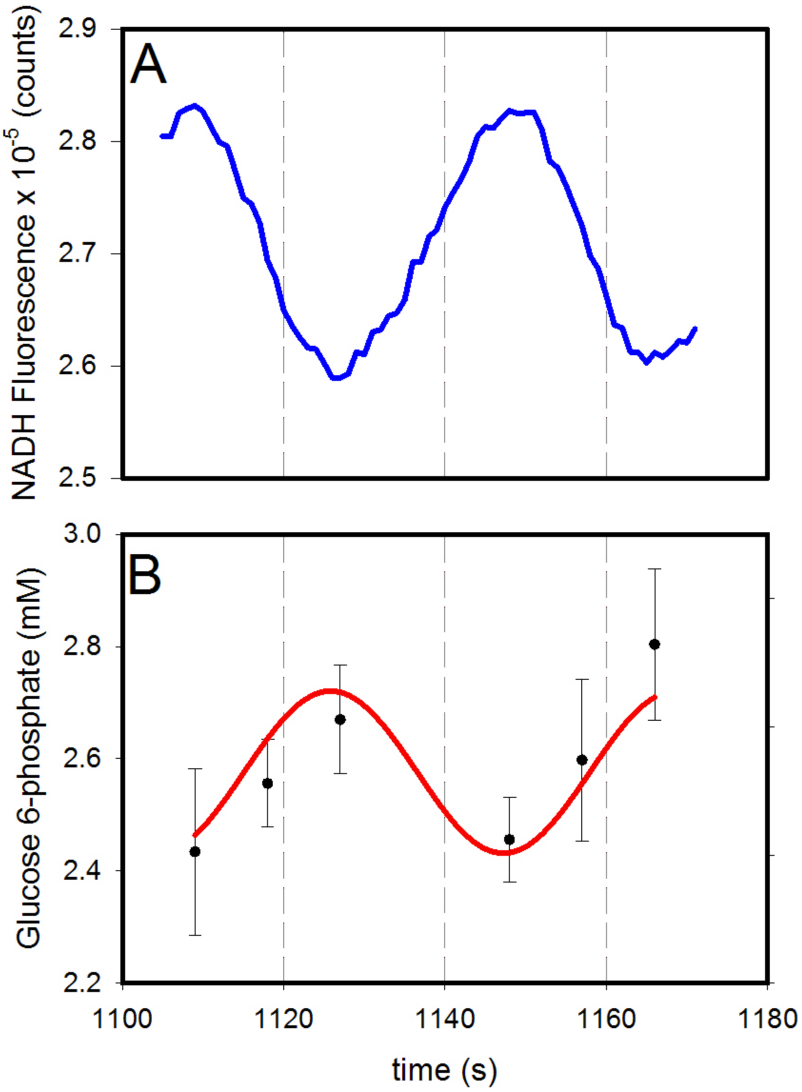

Figure S2 Oscillations in NADH and Glucose 6-phosphate in *S. cerevisiae* cells grown at 30 °C. Yeast cells (10% w/v) were suspended at 25 °C in 100 mM potassium phosphate, pH 6.8 and oscillations were induced as described in Fig. S1. Measurements of intracellular glucose 6-phosphate were made by quenching the cells with boiling buffered ethanol and subsequently extracting the metabolites (13). The concentration of glucose 6-phosphate was then measured by addition of $NADP^+$ and glucose 6-phosphate dehydrogenase to the extract. The concentration of glucose 6-phosphate in the extract was determined from the concentration of formed NADPH using standard curves measured on solutions with known concentrations of glucose 6-phosphate. In the estimation of the intracellular concentration of glucose 6-phosphate we assumed that 1 mg protein corresponds to a cytoplasmic volume of 3.7 µl.

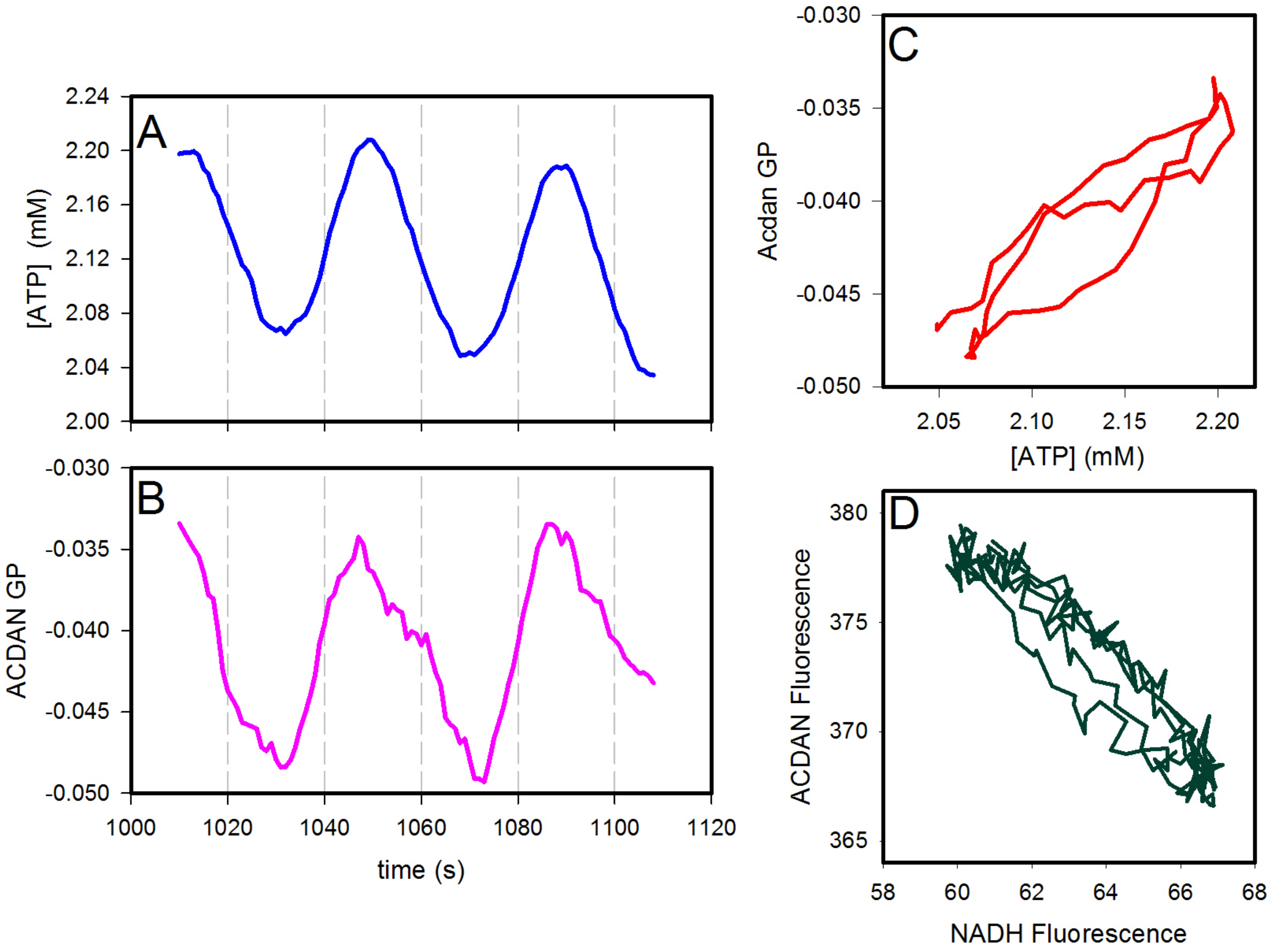

Figure S3 Oscillations in intracellular ATP and ACDAN GP in *S. cerevisiae* cells grown at 30 °C. (A) and (B) time series of ATP and ACDAN GP; (C) phase plot of ACDAN GP versus intracellular ATP; (D) phase plot of ACDAN fluorescence against NADH fluorescence. Yeast cells (10% w/v) were suspended at 25 °C in 100 mM potassium phosphate, pH 6.8 and oscillations were induced as described in Fig. S1 Intracellular ATP concentration was measured using an aptamer-based ATP nanosensor (14) inserted into the cells using electroporation.

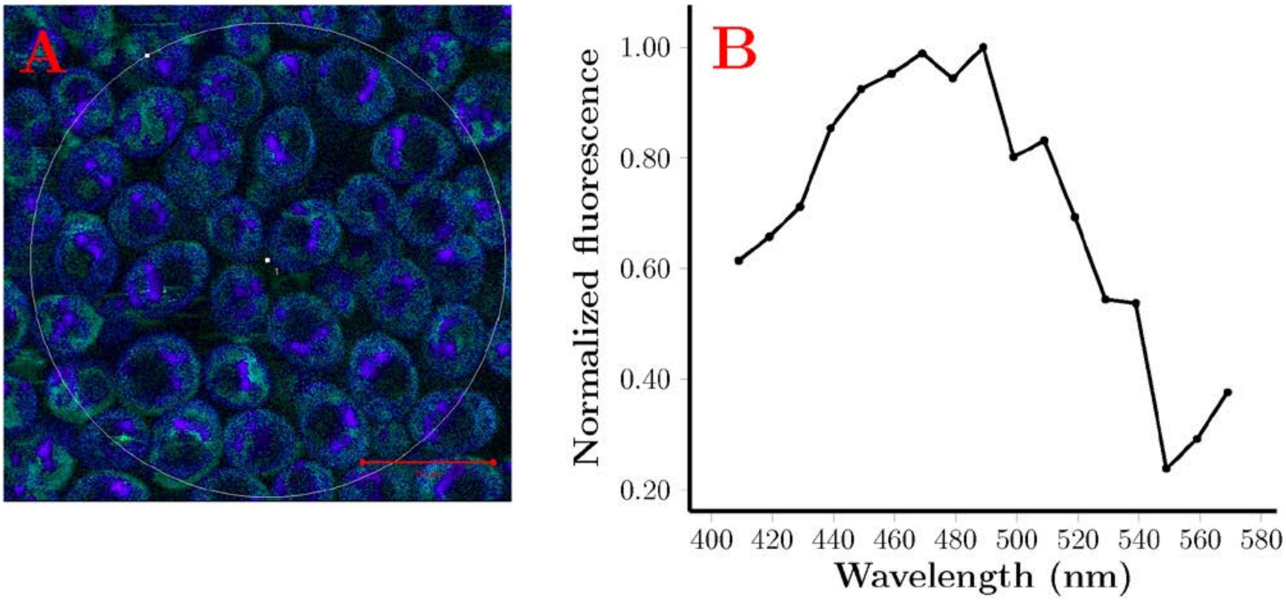

Figure S4 Distribution of ACDAN in resting BY4743 yeast cells. A) Spectral fluorescence image of ACDAN were obtained on an inverted multiphoton excitation fluorescence microscope (Zeiss LSM 510 META NLO, Carl Zeiss, Jena, Germany) equipped with a Ti:Sa MaiTai XF-W2S laser (Broadband Mai Tai with 10 W Millennia pump laser, tunable excitation range 710–980 nm, Spectra Physics, Mountain View, CA), with excitation wavelength for ACDAN at 780 nm. The fluorescence signal was collected using the microscope's meta detector in spectral mode acquisition in a wavelength range from 400 to 580 nm with a resolution of 10 nm, through a 63X water immersion objective, NA 1.2. Acquisition of the two photon excitation fluorescence image was performed by adding 300 µl of ACDAN-labeled resting yeast cells at a density of 10% by weight (suspended in 100 mM potassium phosphate buffer, pH 6.8) to an 8-well plastic chamber (Lab-tek Brand Products, Naperville, IL). The chamber containing the sample was placed in the microscope and the images were acquired a few minutes later to allow the cells to sink to the bottom of the well. B) 10 nm resolution spectra of the encircled region in A. The average fluorescence emission spectrum indicates a heterogeneous and wide distribution of the probe in the intracelullar environment.

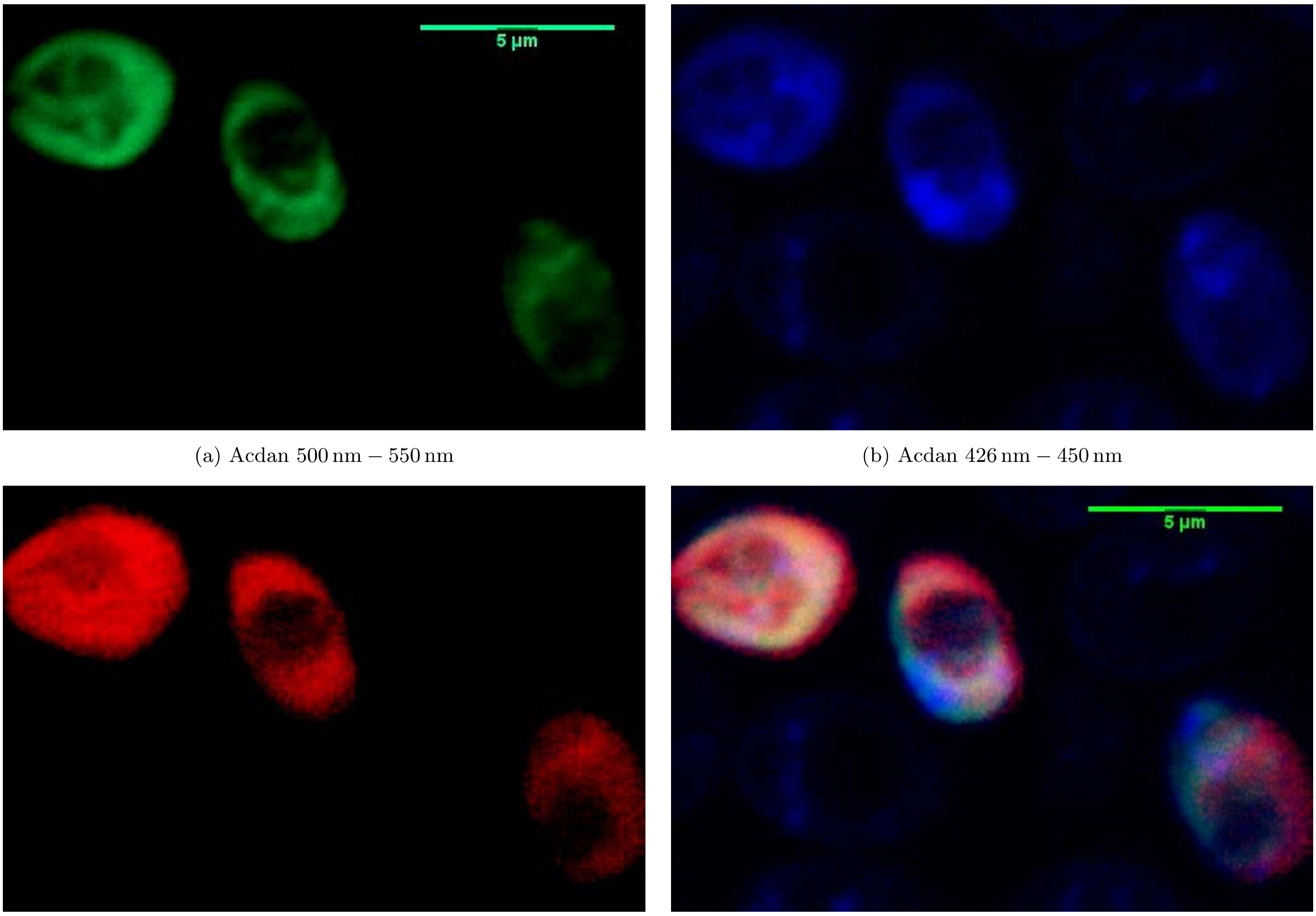

(a) Acdan 500 nm − 550 nm

(b) Acdan 426 nm − 450 nm

(c) CFDA 500 nm − 550 nm

(d) Composite

Figure S5 Distribution of ACDAN and CFDA-SE ester in resting BY4743 cells. BY4743 wild type cells were incubated with 10μM ACDAN and 4.5μM CFDA-SE ester for 1 hour at 30°C, and washed twice with phosphate buffer. (a) and (b) Fluorescence images of ACDAN were obtained using the same microscope (but with different setup) and sample preparation as in Fig. S4. Two photon excitation was performed at 810 nm. The fluorescence emission was collected through a 63X oil objective, NA 1.4 and split to two detectors (H7422 PMT, Hammamatsu, Denmark) by a 460 nm long pass dichroic mirror and then filtered by (a) a 520 ± 17.5 nm band pass filter; (b) 438 ± 12 nm band pass filter (both from AHF Analysen Technik AG, Tübingen, Germany). (c) CFDA was excited via one photon excitation using an Argon-Ion laser at 488nm, send through the confocal pinhole (z-resolution 2μm) and filtered as in (a). (d) Composite of (a), (b) and (c) shows that ACDAN and CFDA co-localize in the cells. The fluorescent images for both dyes were simultaneously obtained using a multi-track mode from the Zeiss software, to avoid leakage of fluorescence into the different emission channels. Control experiments with cells unlabeled and stained with either ACDAN or CFDA confirmed that no leakage was observed within the experimental setup used in the microscope. Scale bar: 5 μm.

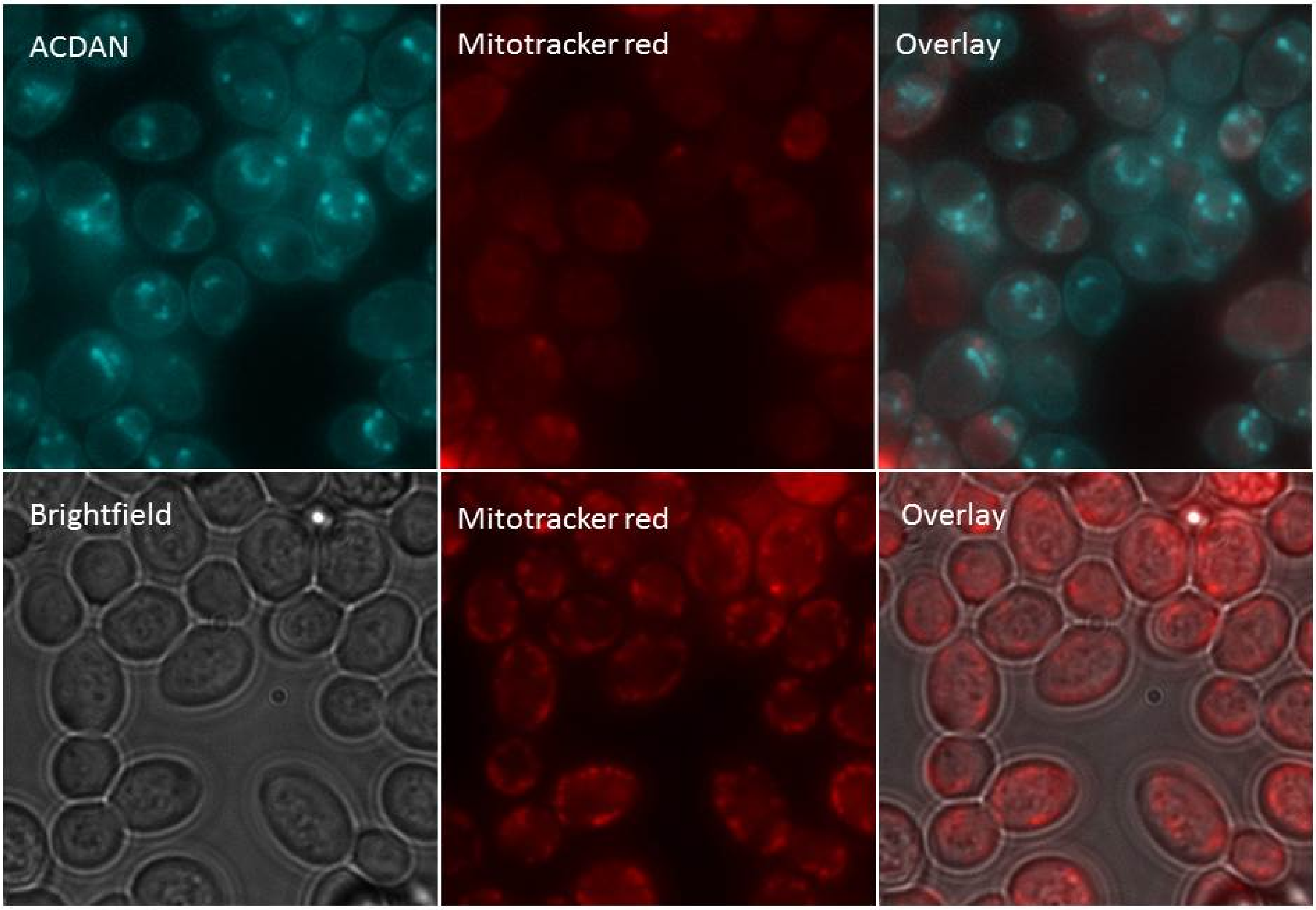

Figure S6 ACDAN and MitoTracker Red in yeast cells. Top row: BY4743 wild type cells grown overnight in glucose and stained with 5 μM ACDAN and 1 μM MitoTracker Red. 10mM glucose was added to the cells prior to imaging. Left, ACDAN; middle, MitoTracker Red; right, overlay. Bottom row: BY4743 wild type cells grown on glycerol and stained with 1 μM MitoTracker Red. Left, brightfield image; middle, MitoTracker Red; left, overlay. Note that the cells grown on glycerol exhibit far more mitochondria and their respiration rate is more than twice that of cells grown on glucose. Images were obtained on a Leica DMRE epifluorescence microscope using a CoolLed illumination system (CoolLed, Andover, U.K.) through a 100X Leica oil-emission objective (NA = 1.4). Images of ACDAN fluorescence were obtained using a Leica Microsystems A4 filter cube, while images of MitoTracker Red were obtained using a Leica Microsystems Y3 filter cube.

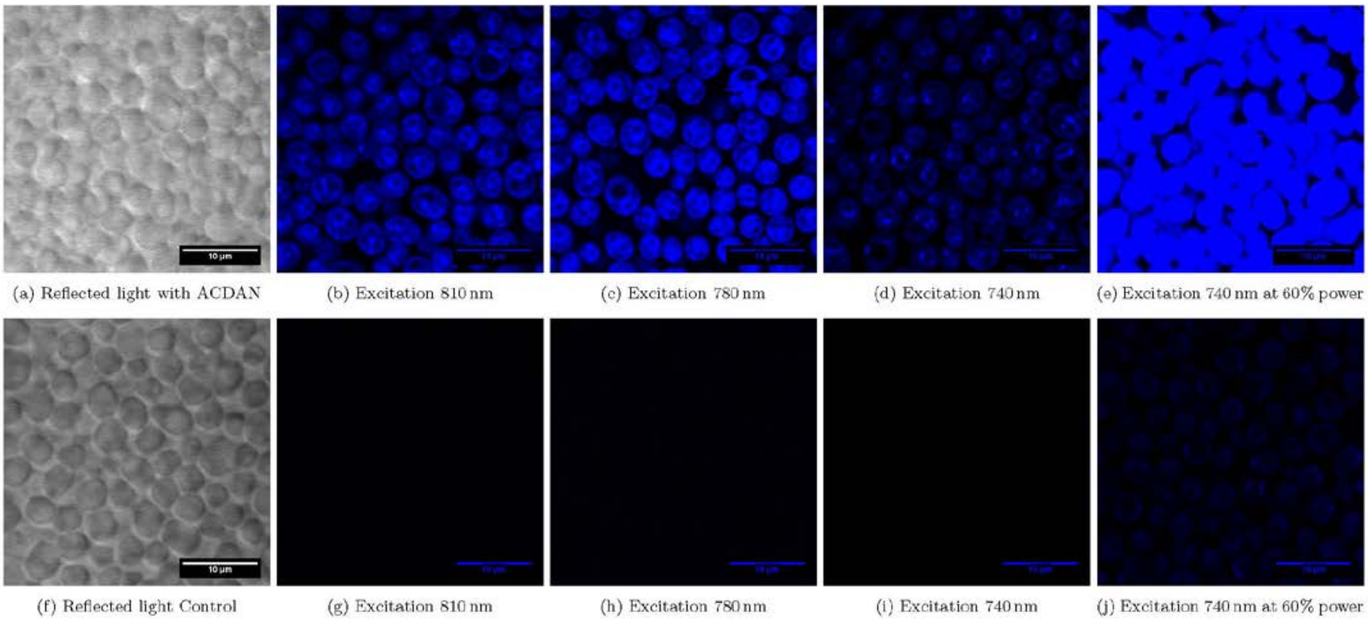

Figure S7 Differences in ACDAN and NADH signal.
Fluorescent images were obtained using the same microscope (with a different setup) as in figure S4. Top row: Cells incubated with ACDAN (10% by weight suspended in 100 mM potassium phosphate buffer with 30 mM glucose, pH 6.8) were excited at either 740nm, 780nm or 810nm, and emission was collected through a 63X oil objective, NA 1.4. (a) are reflected excitation light at 780nm from ACDAN stained cells. (b)-(d): Fluorescence was collected through a 462 ± 24 nm band pass filter (AHF Analysen Technik AG, Tübingen, Germany), which correspond to the fluorescence maxima of NADH and ACDAN upon excitation at 810nm (b), 780nm (c), 740 nm (d) and 740 nm at 60% laser power (e). (f)-(j): Unstained control cells (10% by weight suspended in 100 mM potassium phosphate buffer with 30 mM glucose, pH 6.8) were excited using the same microscope, detector and laser settings as in the top row. No visible differences are observed in the reflected light (f). In the emission range 462 ± 24 nm there is no visible contribution of NADH fluorescence from the control cells upon excitation at 810 (g), 780nm (h) or 740nm (i) at the used settings. Intensity profiles reveal that the intensities in (g)-(i) are not above the background noise. A comparison of (j) and (e) reveals that in order to visualize NADH in unstained cells (j) the ACDAN signal becomes significantly oversaturated (e). The maximum laser power (i.e., the power output from the laser before interaction with filters and optics) are 790mW at 810nm, 740mW at 780nm and 675mW at 740nm. In (b)-(d) and (g)-(i) the used laser power was 40% of the maximum, while in (e) and (j) it was 60%. Scale bars are 10 µm.

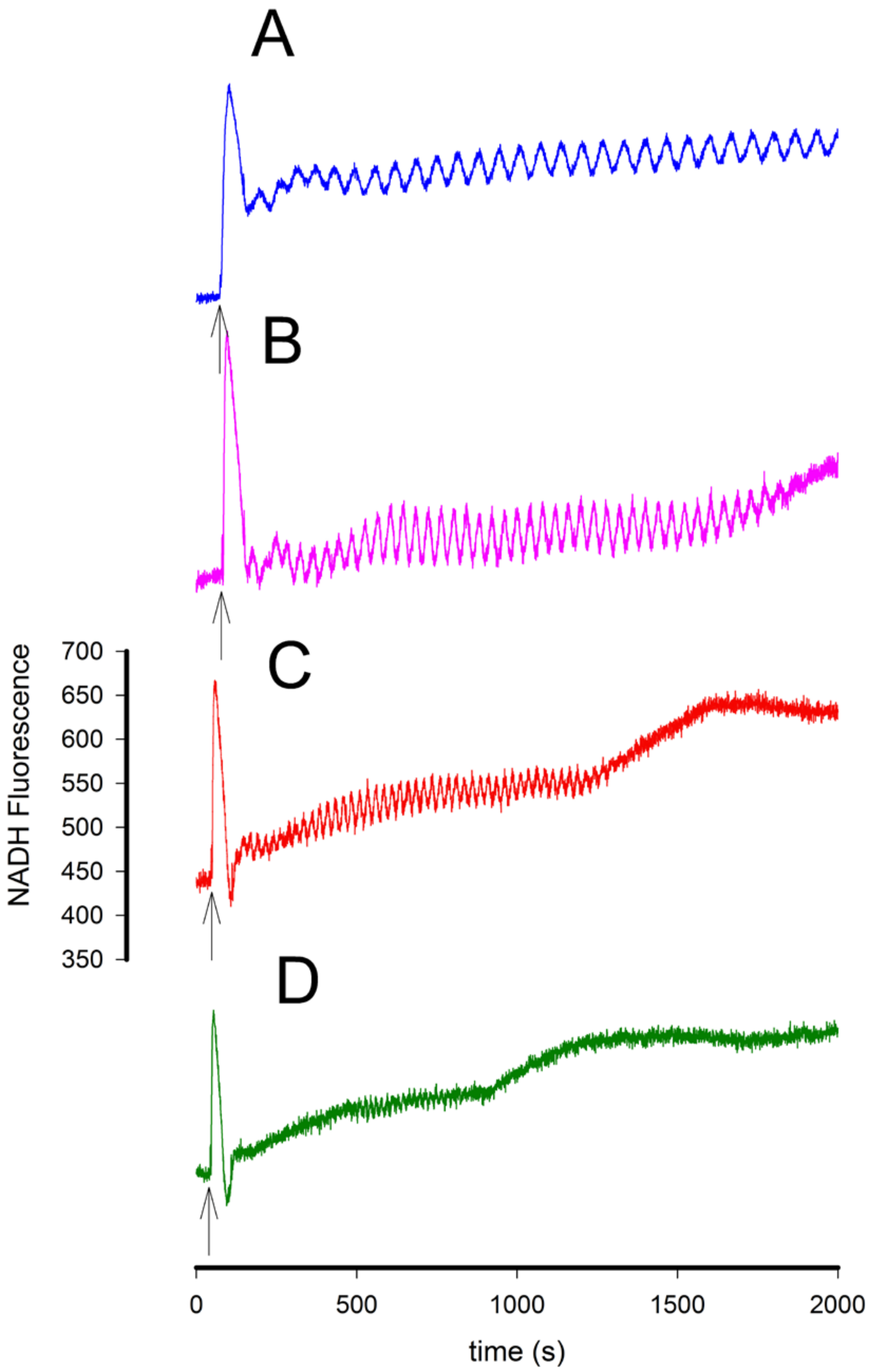

Figure S8. Oscillations of NADH following addition of glucose and KCN to a 10% (w/v) suspension of the *S. cerevisiae* strain BY4743 at (A) 20 °C, (B) 25 °C, (C) 30 °C and (D) 35 °C. Yeast cells were grown at 30 °C. Oscillations were induced by addition of first 30 mM glucose (arrow) and 60 s later 5 mM KCN.

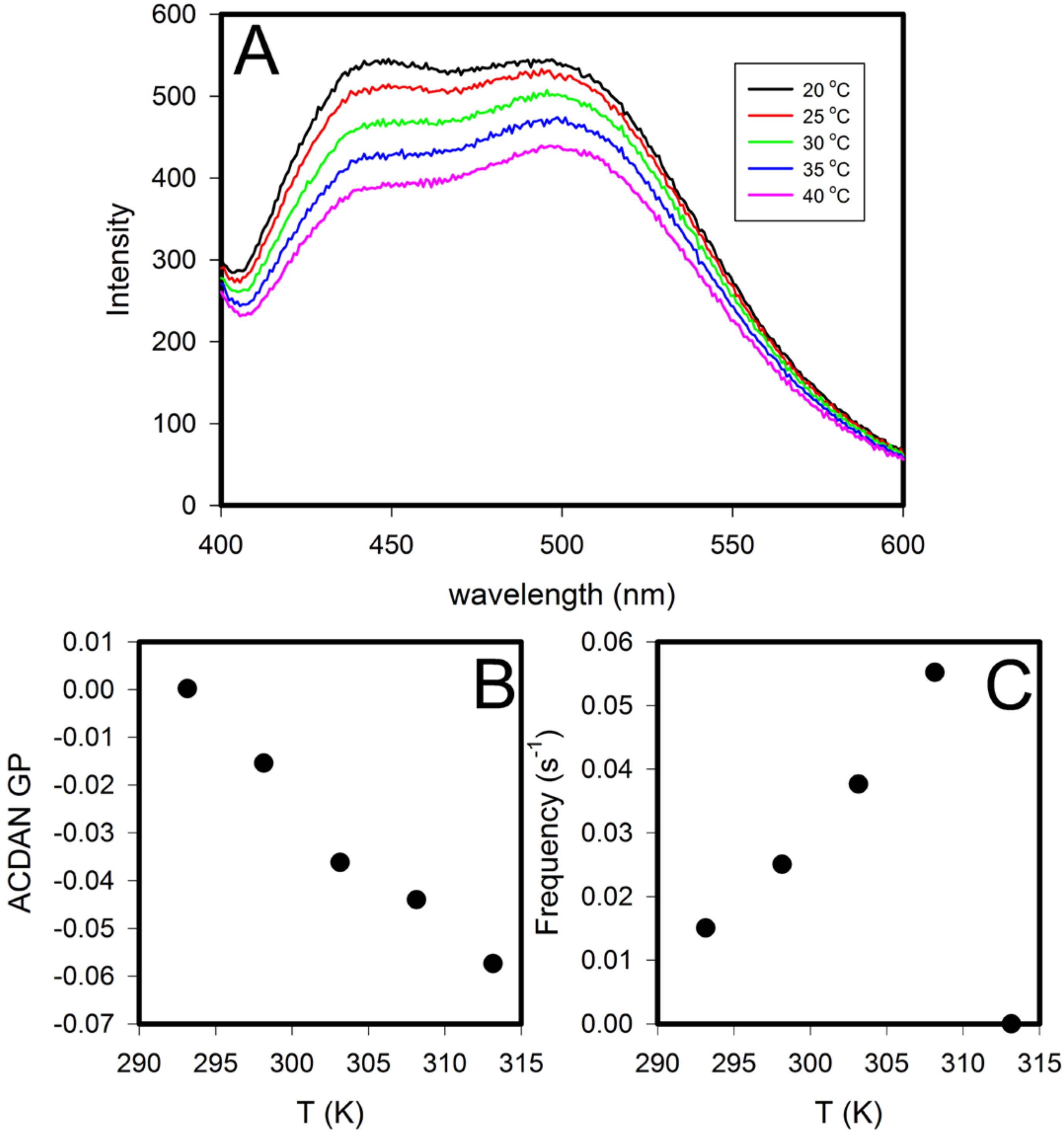

Figure S9. Fluorescence spectra of resting yeast cells incubated with ACDAN and plots of GP and oscillation frequency versus temperature. (A) Fluorescence spectra of 10% (w/v) *S. cerevisiae* strain BY4743 incubated for 60 min with 10 μM ACDAN at room temperature and measured at temperatures of 20, 25, 30, 35 and 40 °C. (B) Plot of GP values, calculated using equation S1 and data from the emission spectra in A, against measuring temperature. (C) Plot of oscillation frequency against measuring temperature. Yeast cells were grown at 25 °C.

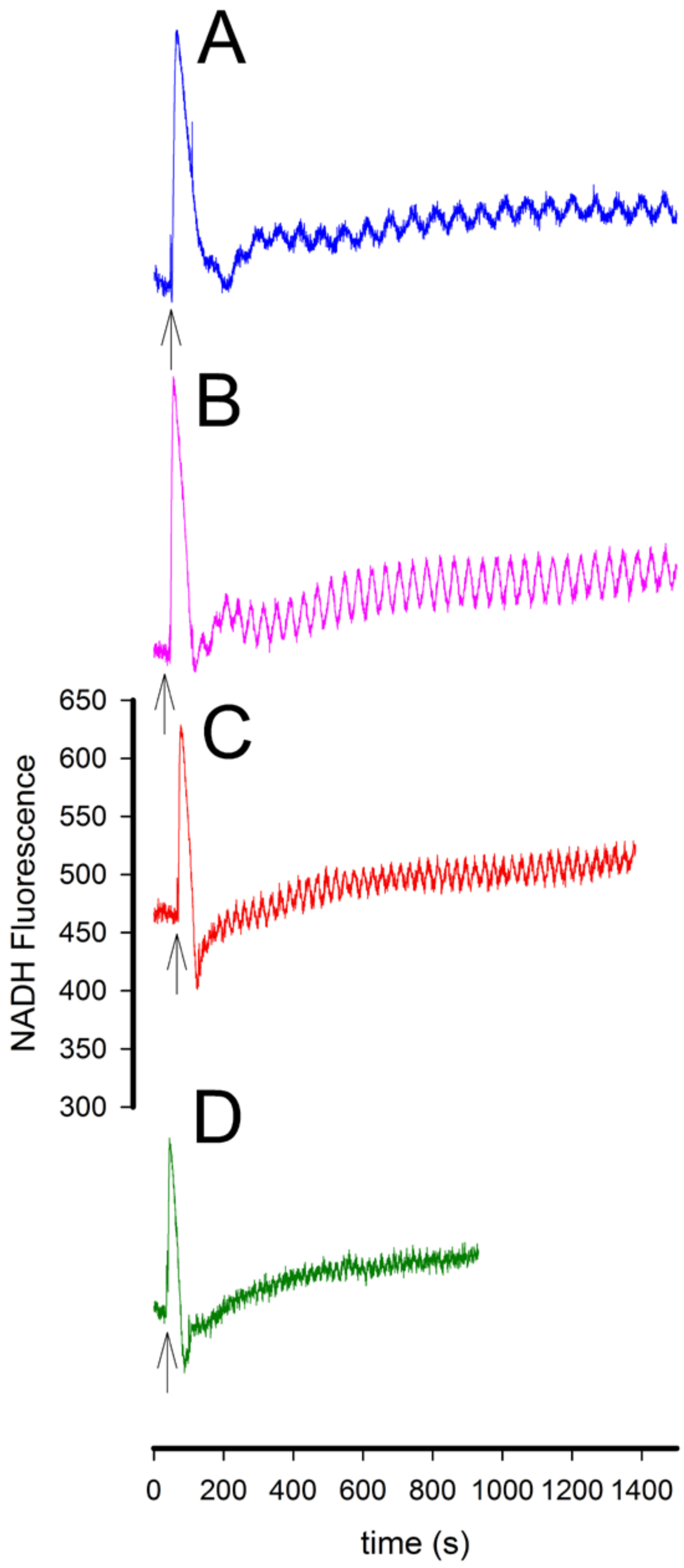

Figure S10. Oscillations of NADH following addition of glucose and KCN to a 10% (w/v) suspension of the *S. cerevisiae* strain BY4743 at (A) 20 °C, (B) 25 °C, (C) 30 °C and (D) 35 °C. Yeast cells were grown at 25 °C. Oscillations were induced by addition of first 30 mM glucose (arrow) and 60 s later 5 mM KCN.

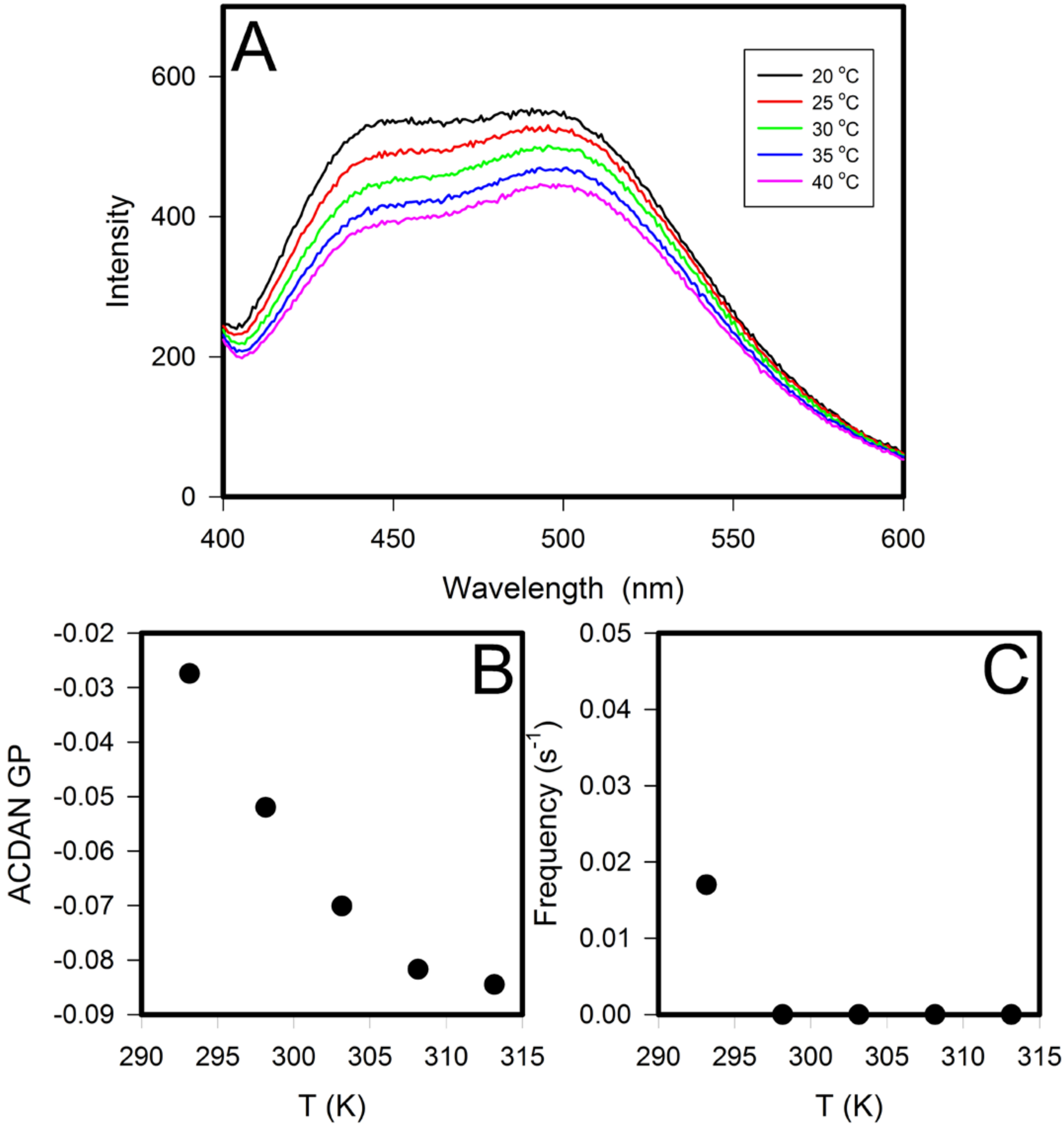

Figure S11. Fluorescence spectra of resting yeast cells incubated with ACDAN and plots of GP and oscillation frequency versus temperature. (A) Fluorescence spectra of 10% (w/v) *S. cerevisiae* strain BY4743 incubated for 60 min with 10 µM ACDAN at room temperature and measured at temperatures 20, 25, 30, 35 and 40 °C. (B) Plot of GP values, calculated using equation S1 and data from the spectra in A, against measuring temperature. (B) Plot of oscillation frequency against measuring temperature. Yeast cells were grown at 35 °C.

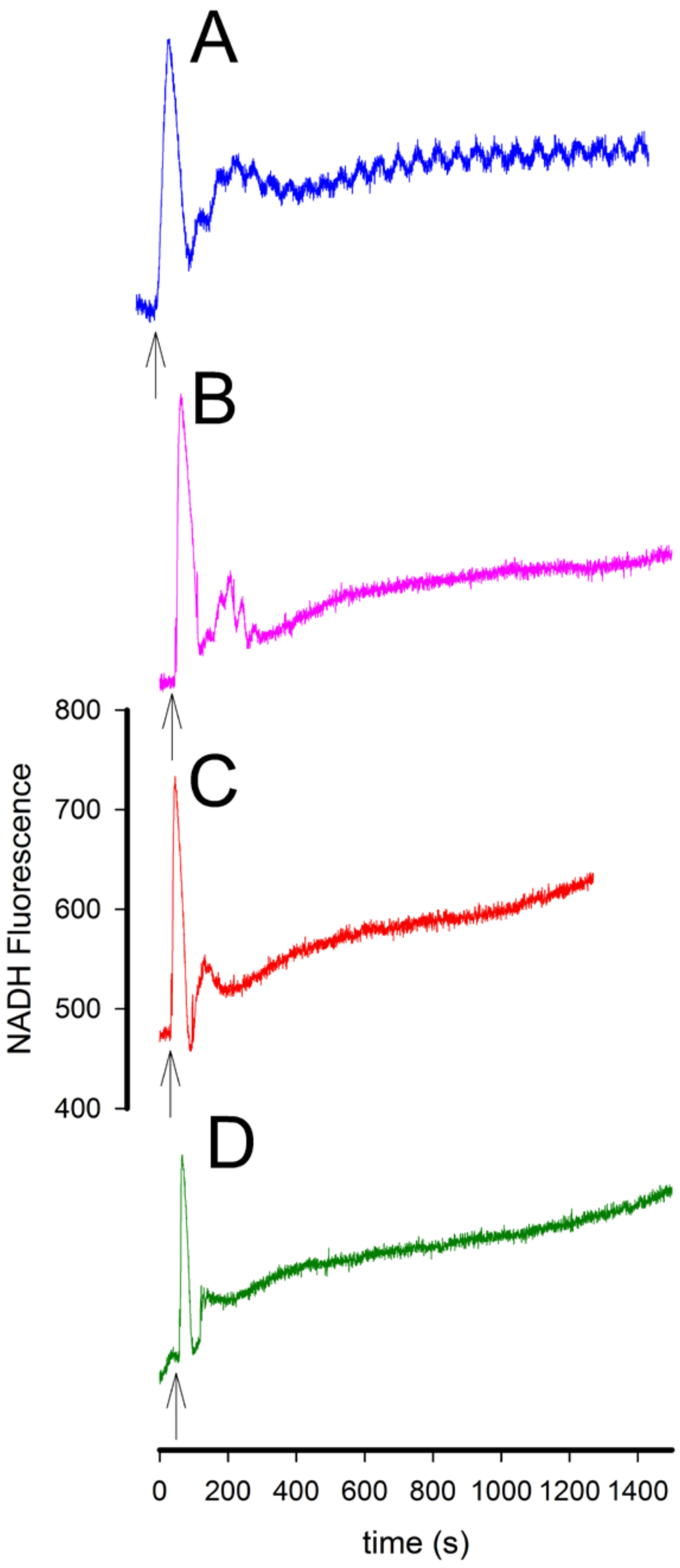

Figure S12. Oscillations of NADH are only observed at 20ºC in yeast cells grown at 35 °C. 30 mM glucose (arrow) and 60 s later 5 mM KCN were added to a 10% (w/v) suspension of the S. cerevisiae strain BY4743 at (A) 20 °C, (B) 25 °C, (C) 30 °C and (D) 35 °C.

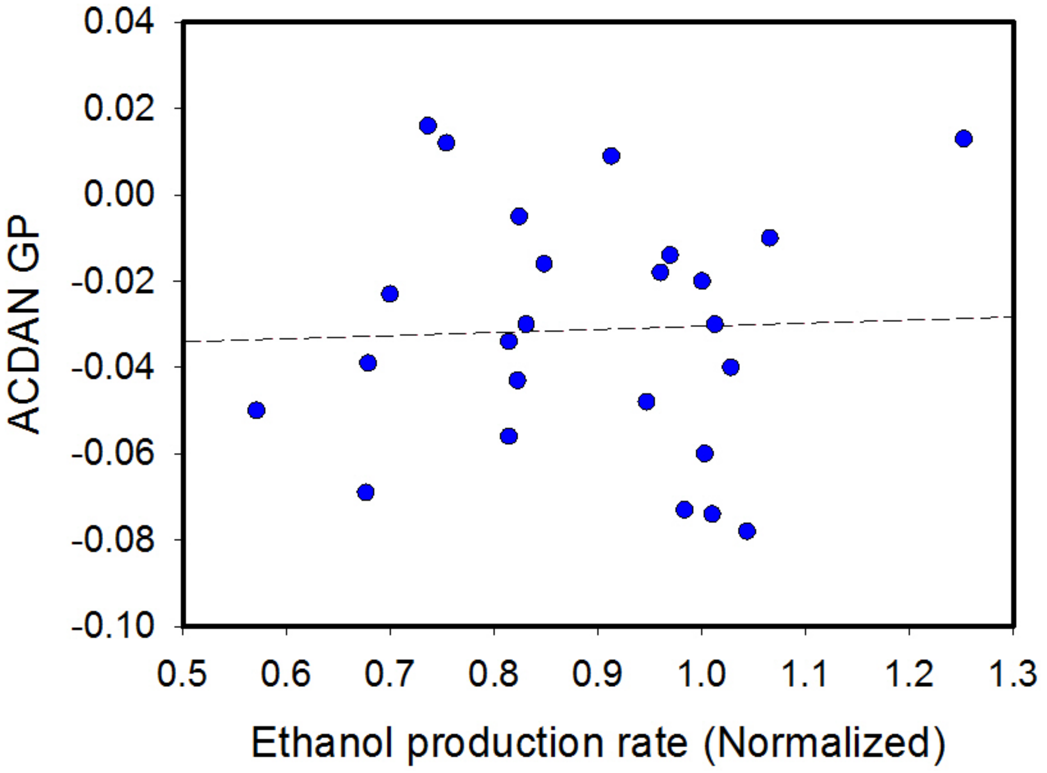

Figure S13. Plot of ACDAN GP vs ethanol production rate in the 25 yeast strains listed in Table S2. The ethanol production rate is normalized to that of the wild type strain. Calibration of the mass spectrometry ethanol signal showed that more than 80% of the added glucose is converted to ethanol in all strains. The dashed line is a linear regression to the data. It has a slope of 7.2×10^{-3} and the R^2 value is 1.6×10^{-3}.

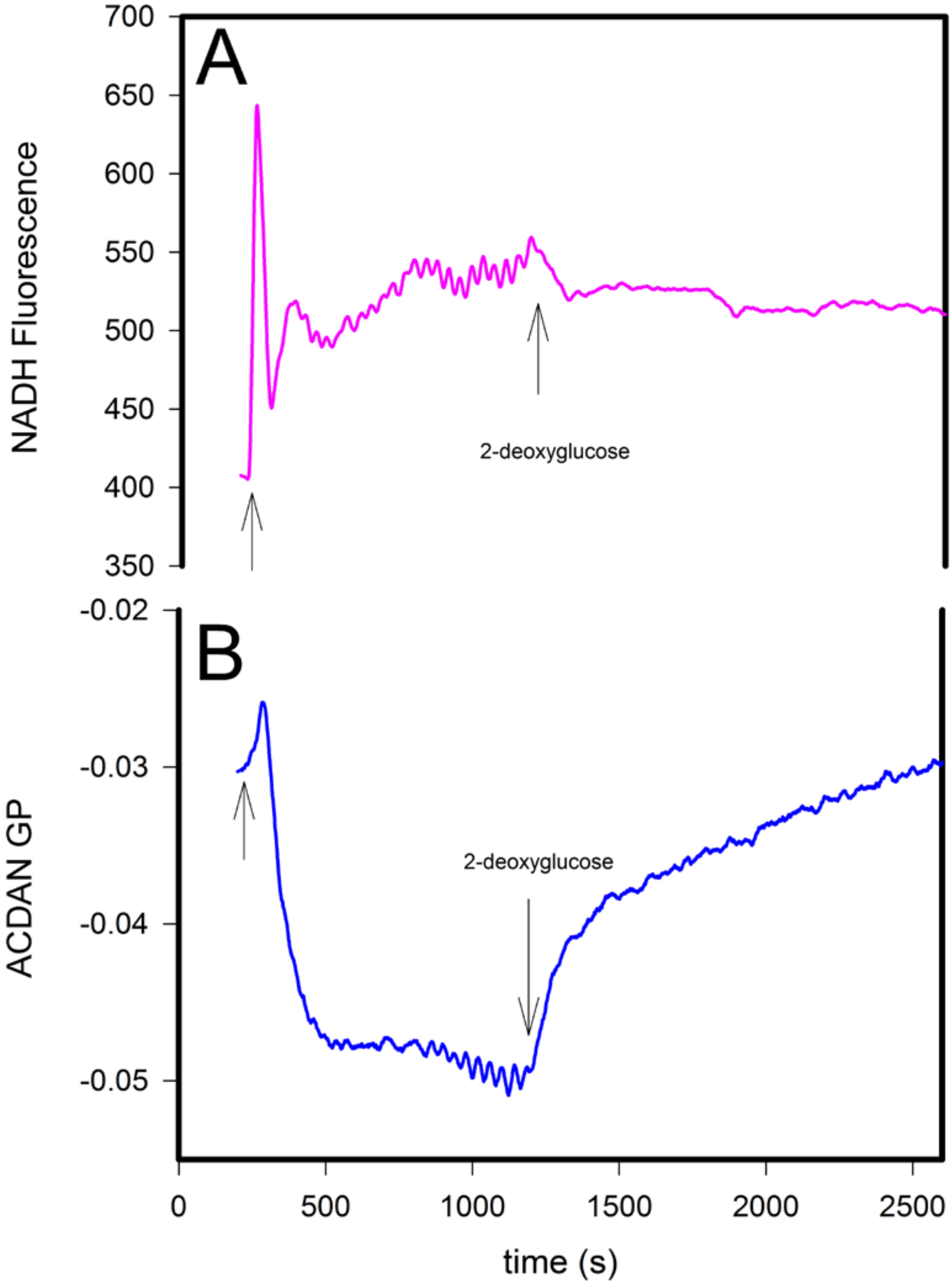

Figure S14. Effect of 2-deoxyglucose on the oscillations of NADH and ACDAN GP. Oscillations in NADH (A) and ACDAN GP (B) following addition of glucose and KCN to a 10% (w/v) suspension of BY4743 WT cells. At approximately 1100 s 20 mM 2-deoxyglucose is added to the suspension. Yeasts were grown at 30 °C. Oscillations were induced by addition of first 30 mM glucose (arrow) and 60 s later 5 mM KCN. Measurement temperature was 25 °C.

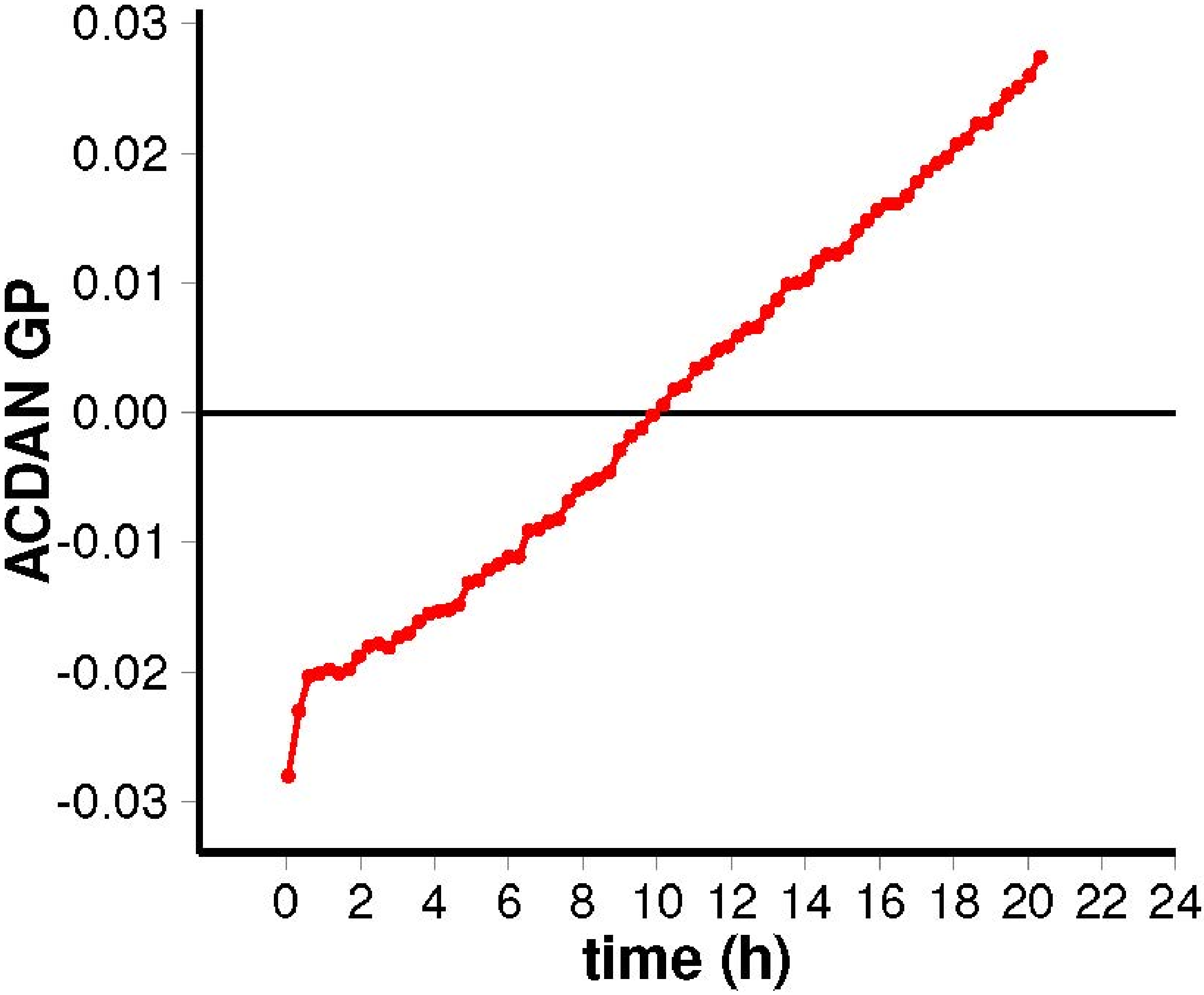

Figure S15. Time evolution of ACDAN GP time in glucose-depleted yeast cells**.** A 10% (w/v) suspension of wild type strain BY4743 grown at 30 °C was incubated with 10 µM ACDAN for 30 mins immediately after glucose in the growth medium had been exhausted. The cells were then washed twice with phosphate/citrate (McIlvaine) buffer (pH = 5.5), and placed in the spectrofluorometer at 30 °C. The fluorescence intensities at 440 nm and 490 nm were recorded simultaneously with a 365nm excitation every 15th minute for approximately 20 hours. A relatively large GP increase in the first hour of starvation is usually observed. Note that the upper GP limit at GP = 0 is crossed after ≈ 10 h, which is consistent with the time for which yeast can be starved before oscillations can no longer be induced.

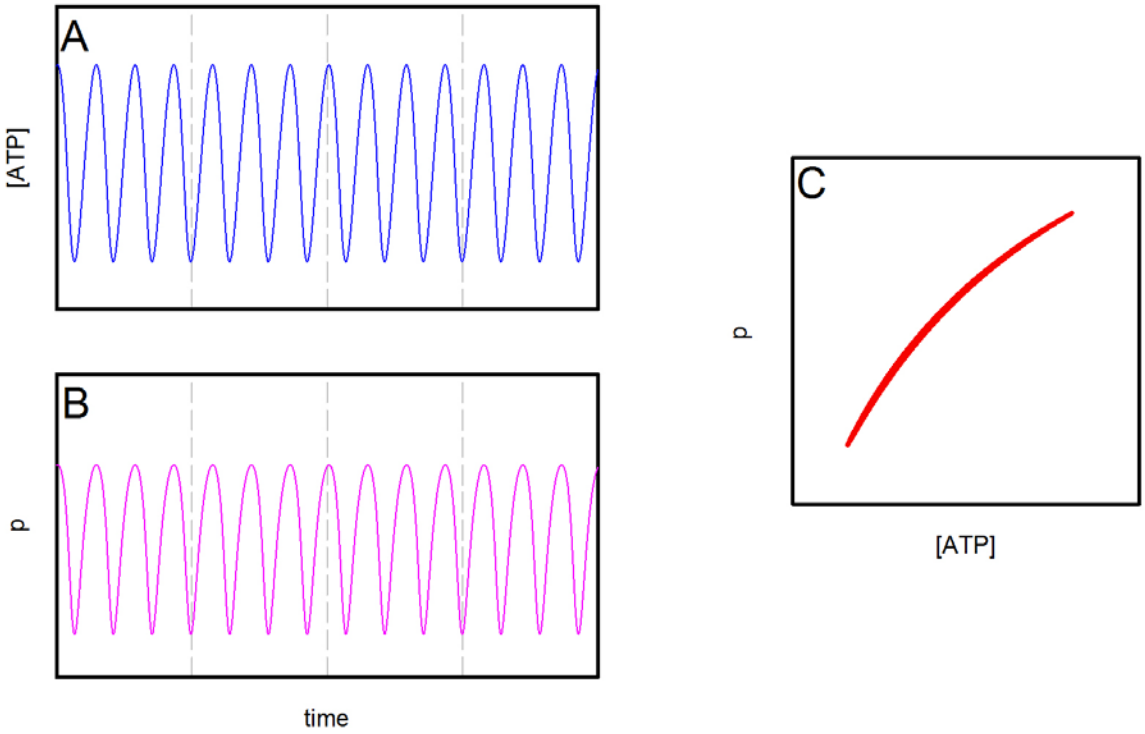

Figure S16 Simulations of the model described by equations S2-S4. (A) shows oscillations in the ATP concentration, while (B) shows oscillations in the polarity (p) of intracellular water. (C) is a phase plot of p against [ATP]. Note the similarity of (C) to the phase plot in Fig. S3C. Parameters (most of them dimensionless) are: v=0.4; V=2.5/p; K_c^{oo} =8·p; $\frac{\gamma_c}{2}$ = 0.5 kcal/mol; K_o^{oo}=50; $\frac{\gamma_o}{2}$ = 0.6 kcal/mol; k_1= 0.07; V_1= 2.3; κ_c^{00} =10; $\frac{\Gamma}{2}$ =0.7 kcal/mol; K_{c1}^{oo}=0.3; $\frac{\gamma_c}{2}$ = 0.5 kcal/mol. Initial values of [ATP], [ADP] and p are 10, 4 and 1.13, respectively

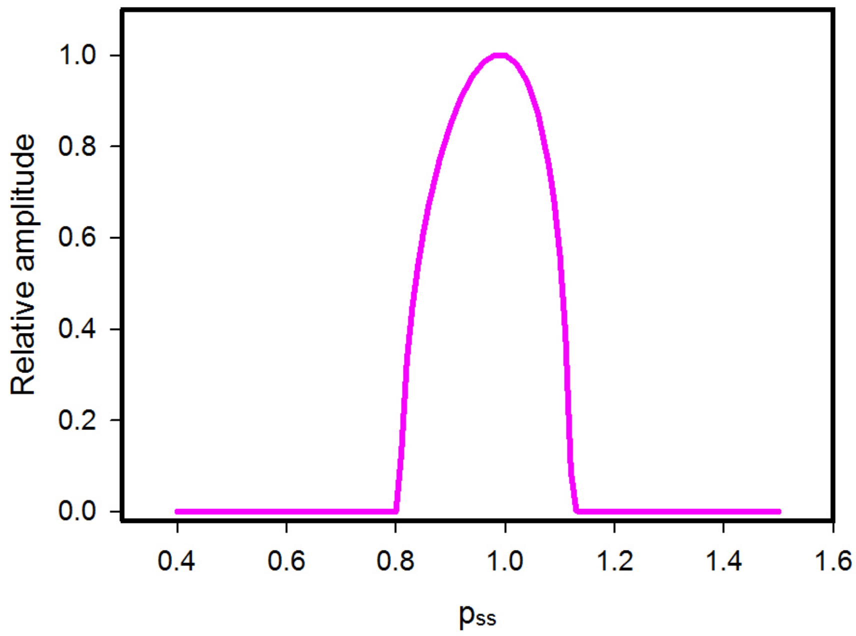

Figure S17 Plot of the relative amplitude of ATP oscillations versus the steady state value of p obtained by setting $\frac{d(p)}{dt}$ equal to zero. Parameters as in Figure S15.

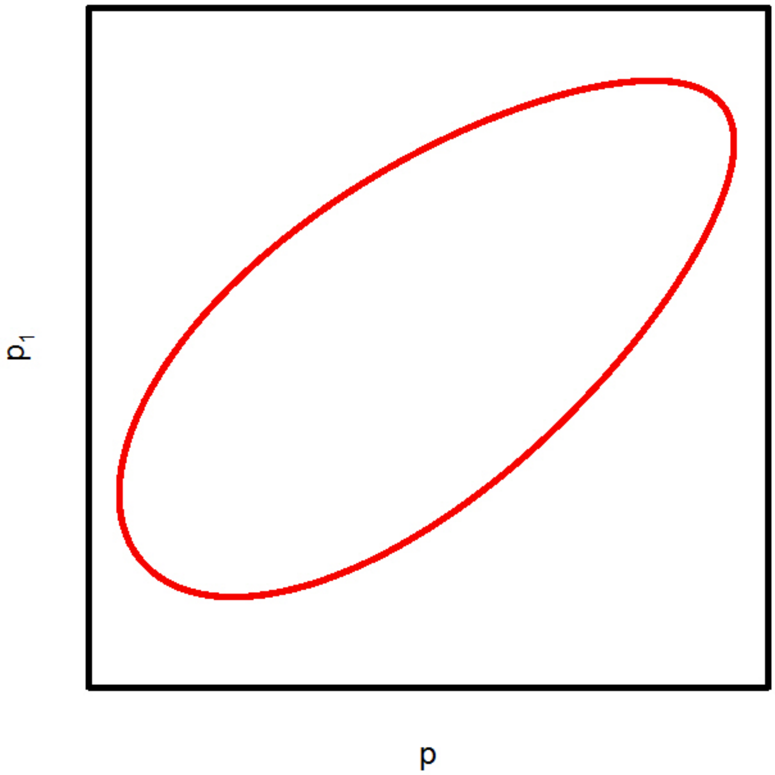

Figure S18 Phase plot of polarization of water in two different regions of the cell: one in which glycolytic oscillations occur (p) and another in which glycolytic oscillations are absent (p_1). The simulation assumes simple mechanical coupling of p to p_1. Parameters as in Fig. S15.

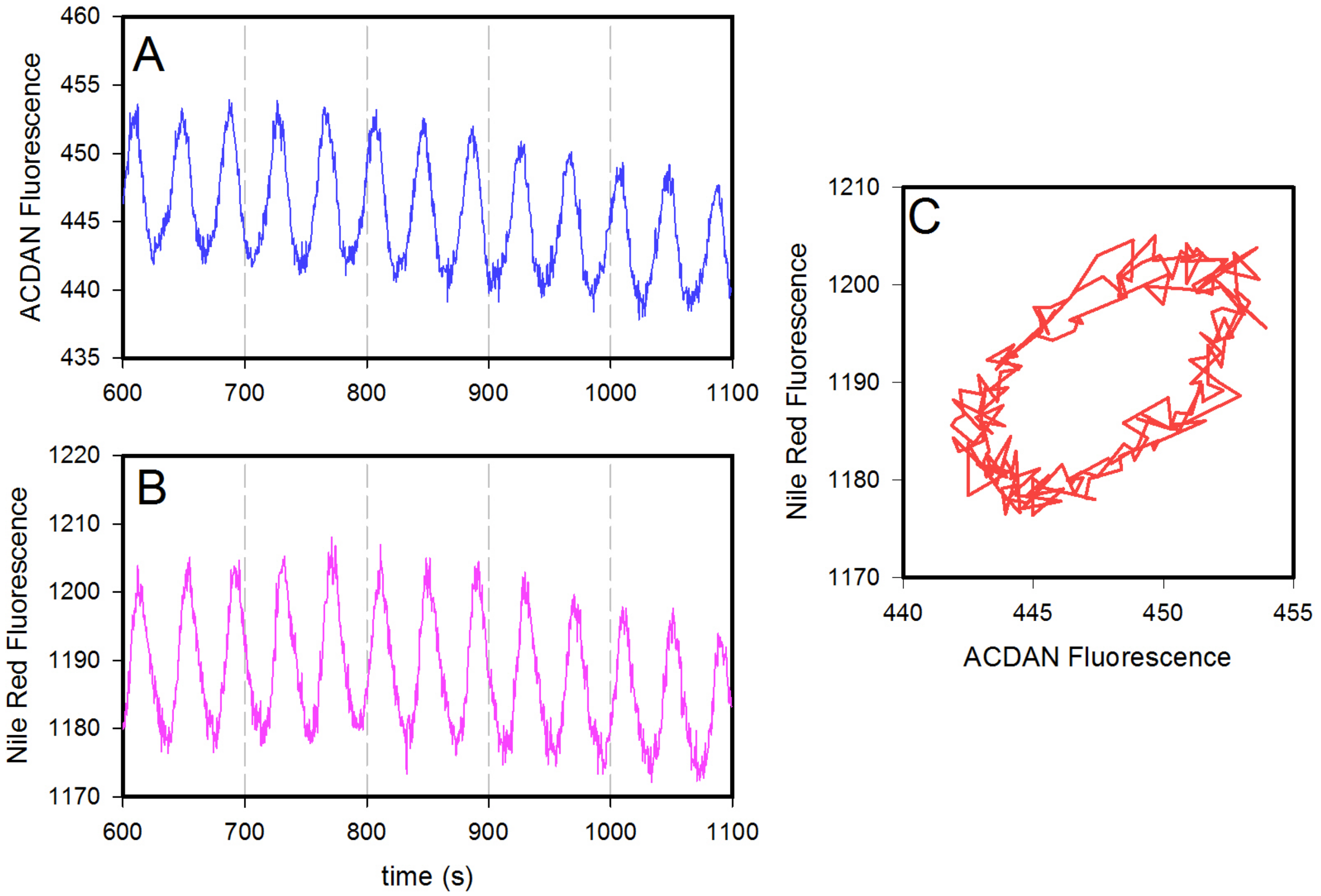

Figure S19 Simultaneous measurements of oscillations in ACDAN (A) and Nile Red (B) fluorescence in the wild type strain *S. cerevisiae* BY4743 strain. A 10% (w/v) cell suspension in 100 mM potassium phosphate buffer, pH 6.8, was incubated at room temperature for 1 h with 10 μM ACDAN and 5 μM Nile red. Then the cells were washed twice and resuspended in the same buffer. ACDAN was excited at 365 nm and its emission was measured at 450 nm, while Nile red was excited at 550 nm with its emission measured at 630 nm. The plot in C is a phase plot of the two measurements. Yeasts were grown at 30 °C. Oscillations were induced by addition of first 30 mM glucose (arrow) and 60 s later 5 mM KCN. Measurement temperature was 25 °C.

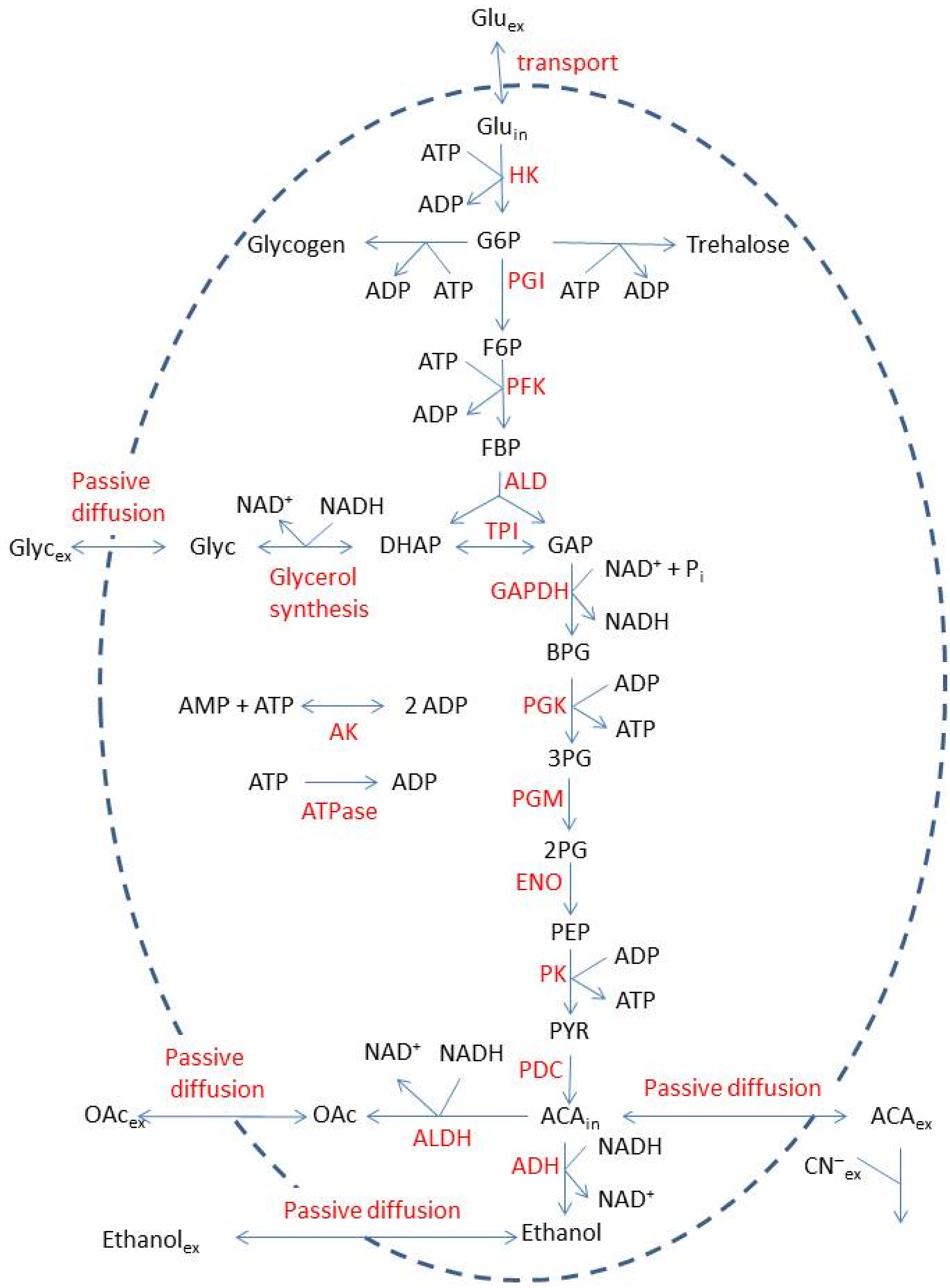

Fig. S20 Hald-Sørensen model (14) of yeast glycolysis. Metabolites are shown in black; enzymes are shown in red. Abbreviations are: Glu$_{ex}$, extracellular glucose; Glu$_{in}$, intracellular glucose; G6P, glucose 6-phosphate; F6P, fructose 6-phosphate; FBP, fructose 1,6-bisphosphate; DHAP, dihydroxyacetone phosphate; Glyc$_{in}$, intracellular glycerol; Glyc$_{ex}$, extracellular glycerol; GAP, glyceraldehyde 3-phosphate; BPG, 1,3-bisphosphoglycerate; 3PG, 3-phosphoglycerate; 2PG, 2-phosphoglycerate; PEP, phosphoenol pyruvate; PYR, pyruvate; ACAin, intracellular acetaldehyde; OAc$_{in}$, intracellular acetate; OAc$_{ex}$, extracellular acetate; ACAex, extracellular acetaldehyde; HK, hexokinase; PGI, phosphoglucose isomerase; PFK, phosphofructokinase; ALD, aldolase; TPI, triosephosphate isomerase; GAPDH, glyceraldehyde phosphate dehydrogenase; PGK, phosphoglycerate kinase; PGM, phosphoglycerate mutase; ENO, enolase; PK, pyruvate kinase; PDC, pyruvate decarboxylase; ALDH; Aldehyde dehydrogase; ADH, alcohol dehydrogenase; AK, adenylate kinase.

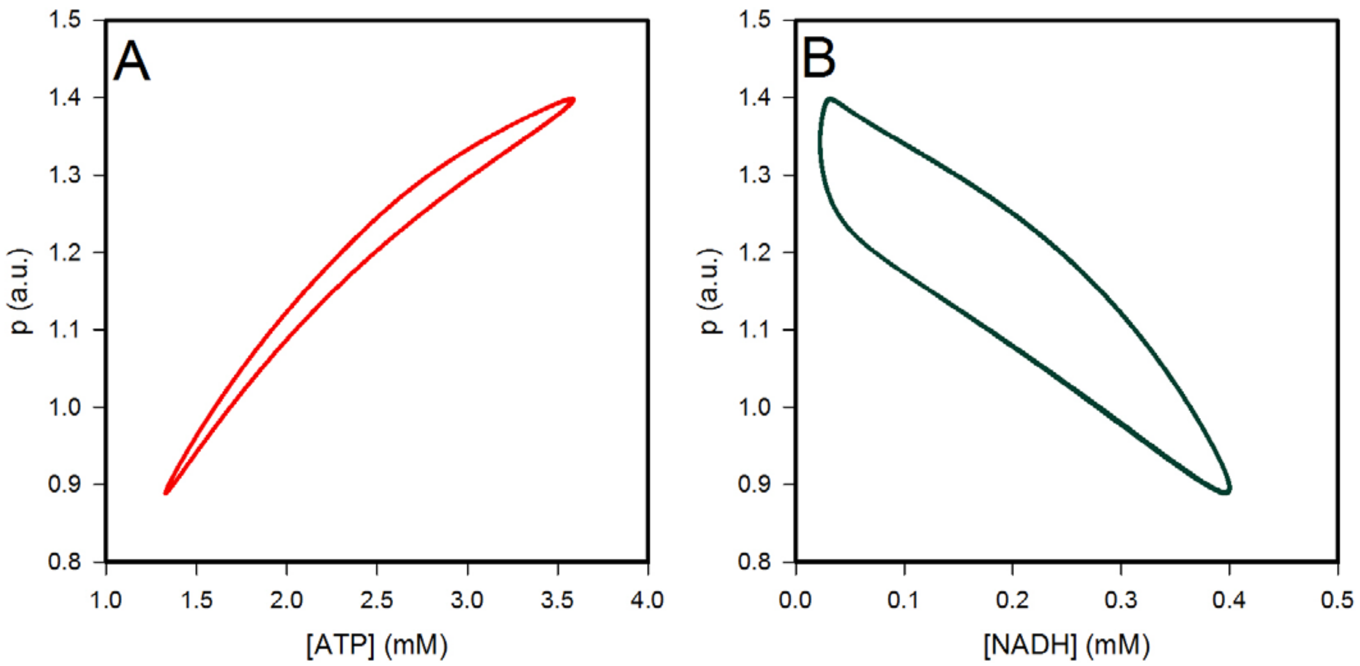

Fig S21 Phase plots of (A) p versus [ATP] and (B) p versus [NADH] for the modified Hald-Sørensen model with inclusion of the polarity of water and effects on maximal enzyme activities and dissociation constants as described for the simple model. The plots should be compared to those in Fig. S3C and S3D.

References

1. Parasassi, T., De Stasio, G., Ravagnan, G., Rusch, R.M. & Gratton, E. Quantitation of lipid phases in phospholipid vesicles by the generalized polarization of Laurdan fluorescence, *Biophys. J.* **60**, 179–189 (1991).

2. Bagatolli L.A. LAURDAN fluorescence properties in membranes: a journey from the fluorometer to the microscope. *Fluorescent Methods to study Biological Membranes,* eds. Mely Y, Duportail G. Springer Series on Fluorescence 13 (series editor M. Hof) 3-35 (Springer, 2013).

3. Bagatolli L.A., Stock R.P. The use of 6-acyl-2-(dimethylamino)naphtalenes as relaxation probes of biological environments. *Perspectives on Fluorescence: A Tribute to Gregorio Weber*, Springer Series on Fluorescence, (ed. Jameson. D.M.) 197-216 (Springer, 2016).

4. Thoke H.S., *et al.* Tight coupling of metabolic oscillations and intracellular water dynamics in Saccharomyces cerevisiae *PLoS ONE* **10**: e0117308; 10.1371/journal.pone.0117308 (2015).

5. Ling, G.N. *Life at the cell and below-cell level*. (Pacific Press, 2001).

6. Goldebeter A. & Lefever R. Dissipative structures for an allosteric model application to glycolytic oscillations**.** *Biophys J* **12**, 1302-1315 (1972).

7. Karreman, G. (1965) Cooperative specific adsorption of ions at charged sites in an electric field. *Bull. Math. Biophys.* **27**, 91-104 (1965).

8. Ling G.N. Diphosphoglycerate and inosine hexaphosphate control of oxygen binding by hemoglobin: a theoretical interpretation of experimental data. *Proc. Natl. Acad. Sci. USA* **67**, 296-301 (1970).

9. Svensson, I., Wehtje E., Adlercreutz, P. & Mattiasson, B. Effects of water activity on reaction rates and equilibrium positions in enzymatic esterifications. *Biotechnol. Bioeng*. **44**, 549–556 (1994).

10. Zimmerman, S.B. & Minton, A.P. Macromolecular crowding: biochemical, biophysical, and physiological consequences. *Ann. Rev. Biophys. Biomol. Struct.* **22**, 27–65 (1993).

11. Murray D.B., Amariei, C. , Sasidharan, K., Machne, R., Aon M.A. & Lloyd, D. Temporal partitioning of the yeast cellular network. In Systems Biology of Metabolic, and Signaling Networks (ed. Aon, M.A. *et al.*) 323-349 (Springer, 2014).

12. Hald B.O., Sørensen, P.G. (2010) Modeling diauxic glycolytic oscillations in yeast. *Biophys. J.*

99(10), 3191 – 3199 (2010).

13. Gonzales, B., Francios, J. & Reneaud, M. A rapid and reliable method for metabolite extraction in yeast using boiling buffered ethanol. *Yeast* **13**, 1347-1356 (1997).

14. Özalp, V.C., Nielsen, L.J. & Olsen, L.F. An aptamer-based nanobiosensor for real-time measurements of ATP dynamics. *ChemBioChem* **11**, 2538-2541 (2010).
